# Differential response of digesta- and mucosa-associated intestinal microbiota to dietary insect meal during the seawater phase of Atlantic salmon

**DOI:** 10.1186/s42523-020-00071-3

**Published:** 2021-01-07

**Authors:** Yanxian Li, Leonardo Bruni, Alexander Jaramillo-Torres, Karina Gajardo, Trond M. Kortner, Åshild Krogdahl

**Affiliations:** 1 Department of Paraclinical Sciences, Faculty of Veterinary Medicine, Norwegian University of Life Sciences, Oslo, Norway; 2 Department of Agriculture, Food, Environment and Forestry, University of Florence, Florence, Italy

**Keywords:** Atlantic salmon, Diet, Black soldier fly, Microbiota, Digesta, Mucosa

## Abstract

**Background:**

Intestinal digesta is commonly used for studying responses of microbiota to dietary shifts, yet evidence is accumulating that it represents an incomplete view of the intestinal microbiota. The present work aims to investigate the differences between digesta- and mucosa-associated intestinal microbiota in Atlantic salmon (*Salmo salar*) and how they may respond differently to dietary perturbations. In a 16-week seawater feeding trial, Atlantic salmon were fed either a commercially-relevant reference diet or an insect meal diet containing ~ 15% black soldier fly (*Hermetia illucens*) larvae meal. The digesta- and mucosa-associated distal intestinal microbiota were profiled by 16S rRNA gene sequencing.

**Results:**

Regardless of diet, we observed substantial differences between digesta- and mucosa-associated intestinal microbiota. Microbial richness and diversity were much higher in the digesta than the mucosa. The insect meal diet altered the distal intestinal microbiota resulting in higher microbial richness and diversity. The diet effect, however, depended on the sample origin. Digesta-associated intestinal microbiota showed more pronounced changes than the mucosa-associated microbiota. Multivariate association analyses identified two mucosa-enriched taxa, *Brevinema andersonii* and *Spirochaetaceae*, associated with the expression of genes related to immune responses and barrier function in the distal intestine, respectively.

**Conclusions:**

Our data show that salmon intestinal digesta and mucosa harbor microbial communities with clear differences. While feeding insects increased microbial richness and diversity in both digesta- and mucosa-associated intestinal microbiota, mucosa-associated intestinal microbiota seems more resilient to variations in the diet composition. To fully unveil the response of intestinal microbiota to dietary changes, concurrent profiling of digesta- and mucosa-associated intestinal microbiota is recommended whenever feasible. Specific taxa enriched in the intestinal mucosa are associated to gene expression related to immune responses and barrier function. Detailed studies are needed on the ecological and functional significance of taxa associated to intestinal microbiota dwelling on the mucosa.

**Supplementary Information:**

The online version contains supplementary material available at 10.1186/s42523-020-00071-3.

## Background

The global population is projected to reach 9.7 billion in 2050 [1], requiring an increase in the food supply by 25–70% [2]. Producing more safe and high-quality food in a sustainable way to meet the global population growth is a great challenge for our generation. Fish are considered as nutritionally valuable part of the human diet and play an important role in the global food supply [3, 4]. The average annual growth rate of world food fish consumption in the period 2019–2030 is projected to be 1.4%, reaching 28 million tonnes live weight in 2030 [5]. Atlantic salmon (*Salmo salar*) is the most produced marine fish species and one of the most economically important farmed fish worldwide [6]. While Atlantic salmon are strictly carnivorous in the wild, farmed Atlantic salmon have experienced a substantial shift in the diet composition due to a limited supply of marine ingredients. Marine ingredients used for Norwegian farmed Atlantic salmon have gradually been replaced by plant sources, decreasing from ~ 90% in 1990 to ~ 25% in 2016 [7]. Due to concerns on the economic, environmental and social sustainability of the current raw materials for Atlantic salmon farming [6], more sustainable alternative feed ingredients, such as insects [8] and single-cell organisms (bacteria, yeasts and microalgae) [9], have been developed and used. One of the insect species with the greatest potential as an alternative feed ingredient for salmon aquaculture is black soldier fly (BSF; *Hermetia illucens*), which is now produced at industrial scale in Europe. In terms of protein quality, BSF larvae have a favorable essential amino acid profile closer to fishmeal than that of soybean meal [10]. The nutritional value of BSF larvae meal has been extensively evaluated in various fish species including Atlantic salmon [11–21]. However, how dietary BSF larvae meal may influence the intestinal health, function and microbiota of fish remains largely unexplored.

It is now well established that intestinal microbiota plays a pivotal role in host development and physiology, from being an essential element for the development of normal gut functions and immunity [22, 23] to modulating lipid metabolism and energy balance [24, 25]. Recent advances in sequencing technologies have transformed our ability to study the composition and dynamics of fish intestinal microbiota, leading to increasing interest in selective manipulation of intestinal microbiota. Diet is one of the key factors in shaping the intestinal microbiota. While long-term dietary habits have a considerable effect on the structure and activity of host intestinal microbiota [26–28], short-term dietary change also alters the intestinal microbiota in a rapid and reproducible way [29]. Different dietary components selectively promote or suppress the growth of certain microbial clades, which in turn could inflict important effects on the host health and disease resistance [30, 31]. The use of alternative feed ingredients may not only affect the nutrient utilization, fish growth, health, welfare and product quality, but also intestinal microbiota in Atlantic salmon [32–34]. While studies in mammals and fish have revealed substantial differences between the digesta- and mucosa-associated intestinal microbiota [32, 35–38], most studies investigating diet effects on the intestinal microbiota of fish have sampled the digesta only or a mixture of digesta and mucosa. Evidence is accumulating that digesta- and mucosa-associated intestinal microbiota in fish respond differently to dietary changes [32, 39–42]. Profiling only one of or a mixture of digesta- and mucosa-associated microbiota may obscure the response of intestinal microbiota to dietary changes.

Characterizing intestinal microbiota and its associations with host responses is an essential step towards identifying key microbial clades promoting fish health and welfare. Ultimately, a milestone in the fish microbiota research would be knowing how to selectively manipulate the microbiota to improve the growth performance, disease resistance and health status of farmed fish. The main aims of the present study were (i) to compare distal intestinal microbiota of Atlantic salmon fed a commercially relevant diet or an insect meal diet, (ii) to further explore the dissimilarity between digesta- and mucosa-associated microbiota and the differences in their response to dietary changes, and (iii) to identify associations between microbial clades and host responses. This work was part of a larger study consisting of a freshwater and seawater feeding trial that aimed to investigate the nutritional value and possible health effects for Atlantic salmon of a protein-rich insect meal produced from BSF larvae. The results presented herein focus on the intestinal microbiota in seawater phase Atlantic salmon fed an insect meal diet containing ~ 15% BSF larvae meal for 16 weeks.

## Results

To aid readers in interpreting the data we report here, results on the feed utilization, growth performance, fillet quality, intestinal histopathology and gene expression, which have been reported elsewhere [43–45], are summarized as the following. In brief, there was lack of evidence that the insect meal diet negatively affected the feed utilization, growth performance or fillet quality of Atlantic salmon. Profiling of genes related to lipid metabolism, immune responses, barrier functions and stress responses in the proximal and distal intestine showed little evidence of diet effect. Histopathological examination of intestinal segments showed enterocyte steatosis in the proximal and mid intestine in both diet groups, but it was less severe in the proximal intestine of fish fed the insect meal diet.

Hereafter, different sample groups are named based on the combination of diet (REF vs. IM) and sample origin (DID vs. DIM). Hence, in addition to the extraction blanks, library blanks and mock, we have four different sample types, i.e., REF-DID, REF-DIM, IM-DID and IM-DIM.

### qPCR

Since Cq values of most mucosa DNA templates were out of the linear range of the standard curve, the raw Cq value was used as a proxy of 16S rRNA gene quantity in the diluted DNA templates (Fig. S1). On average, REF-DID showed the highest 16S rRNA gene quantities (mean Cq = 24.7), followed by the mocks (mean Cq = 26.1) and IM-DID (mean Cq = 28.4). Irrespective of diet, mucosa DNA templates (REF-DIM, IM-DIM) showed similar 16S rRNA gene quantities (mean Cq = 30) that were close to extraction blanks (mean Cq = 32.4).

### Characteristics of the sequence data

The high-throughput sequencing generated a total number of 7.5 million raw reads for biological samples. The median of raw reads per sample was 96,231, with the minimum and maximum value being 14,940 and 151,916, respectively. After the sequence denoising and ASV filtering, a total number of 1620 unique ASVs was generated. The mean percentage of chloroplasts and mitochondria removed from the ASV table before filtering out contaminants was 7.4 and 0.2%, respectively. The mean percentage of chloroplast varied considerably between digesta (7.1%) and mucosa (0.2%) samples and between diets within digesta samples, e.g. REF-DID (24.9%) and IM-DID (4.4%). The number of effective sequences retained for the downstream data analysis was 3.6 million. The median of effective sequences per sample was 46,372, with the minimum and maximum value being 951 and 106,591, respectively.

### Taxonomic composition

All the eight bacterial species included in the mock were successfully identified at genus level with *E. faecalis, L. fermentum, L. monocytogenes* and *S. aureus* further being annotated at the species level (Fig. S2A). At the genus level, the average Pearson’s *r* between the expected and observed taxonomic profile of the mock was 0.33, whereas the Pearson’s *r* between the observed taxonomic profile of the mock was 0.98. The relative abundance of most Gram-positive bacteria, *L. monocytogenes* and *E. faecalis* in particular, were underestimated. In contrast, the relative abundance of Gram-negative bacteria was overestimated. Most ASVs (97.5–99.9%) in the extraction and library blanks were classified as *Pseudomonas* (Fig. S2B), which was the main contaminating taxon removed from the biological samples. Other contaminating ASVs removed from the biological samples were classified as *Curtobacterium*, *Jeotgalicoccus*, *Modestobacter*, *Cutibacterium*, *Hymenobacter*, *Brevundimonas*, *Micrococcus*, *Sphingomonas*, *Devosia*, *Sphingomonas aurantiaca* and *Marinobacter adhaerens*. The exact sequence and taxonomy of the contaminating ASVs and their relative abundance in the extraction and library blanks are available in Table S1.

**Fig. 1:**
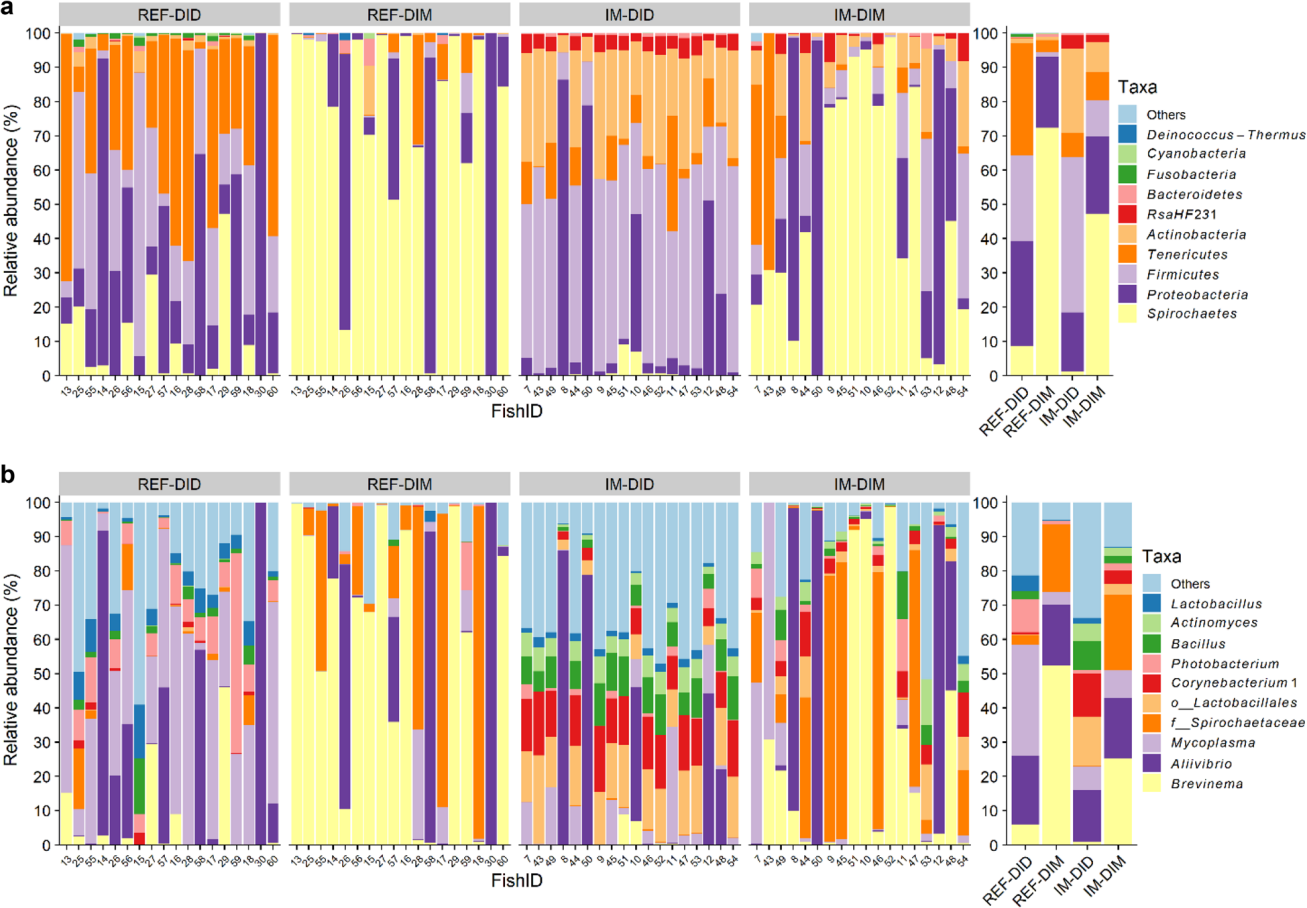
Top 10 most abundant taxa of all samples at phylum (**a**) and genus (**b**) level. The samples are grouped by the sample type. The mean relative abundance of each taxon within the same sample type is displayed on the right side. *o__*, order; *f*__, family; REF, reference diet; IM, insect meal diet; DID, distal intestine digesta; DIM, distal intestine mucosa

The taxonomic composition of mucosa samples showed higher similarity than that of the digesta samples, which were more diet-dependent (Fig. 1). At the phylum level, the dominant taxa of mucosa samples for both diets were *Spirochaetes* (REF-DIM, 72%; IM-DIM, 47%) (mean relative abundance), *Proteobacteria* (REF-DIM, 21%; IM-DIM, 23%), *Firmicutes* (REF-DIM, 1%; IM-DIM, 11%), *Tenericutes* (REF-DIM, 4%; IM-DIM, 8%) and *Actinobacteria* (REF-DIM, 1%; IM-DIM, 9%). For digesta samples, the dominant taxa of REF-DID were *Tenericutes* (33%), *Proteobacteria* (31%), *Firmicutes* (25%) and *Spirochaetes* (9%), whereas IM-DID was dominated by *Firmicutes* (45%), *Actinobacteria* (25%), *Proteobacteria* (17%), *Tenericutes* (7%) and *RsaHF231* (4%) (Fig. 1a). At the genus level, the dominant taxa of mucosa samples for both diets were *Brevinema* (REF-DIM, 52%; IM-DIM, 25%), *Spirochaetaceae* (REF-DIM, 20%; IM-DIM, 22%), *Aliivibrio* (REF-DIM, 18%; IM-DIM, 18%) and *Mycoplasma* (REF-DIM, 4%; IM-DIM, 8%). For digesta samples, the dominant taxa of REF-DID were *Mycoplasma* (33%), *Aliivibrio* (20%), *Photobacterium* (10%), *Brevinema* (6%) and *Lactobacillus* (5%), whereas IM-DID was dominated by *Aliivibrio* (15%), *Lactobacillales* (14%), *Corynebacterium 1* (13%), *Bacillus* (8%), *Mycoplasma* (7%) and *Actinomyces* (5%) (Fig. 1b).

### Core ASVs

**Fig. 2:**
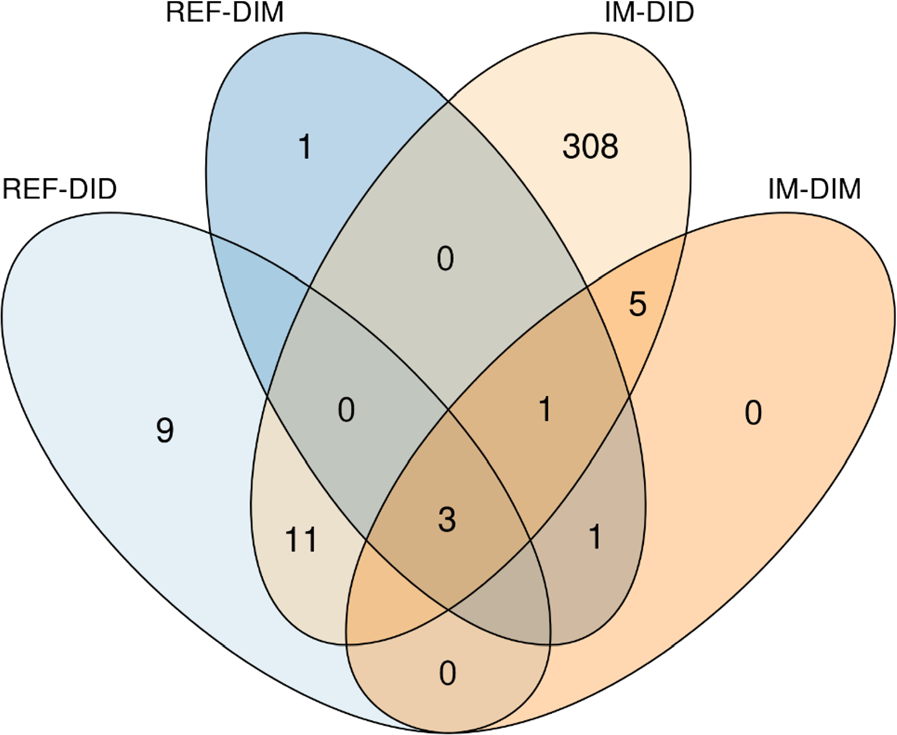
Venn’s diagram showing the shared and unique core ASVs in each sample type. The core ASVs were computed using a prevalence threshold at 80%. REF, reference diet; IM, insect meal diet; DID, distal intestine digesta; DIM, distal intestine mucosa

In total, 339 ASVs were identified as core ASVs based on their prevalence in each sample type (Fig. 2; Table S2). Three ASVs, classified as *Aliivibrio*, *Brevinema andersonii*, and *Mycoplasma* respectively, were identified as core ASVs in all the sample types. The *Brevinema andersonii* ASV was universally present in all the samples. Additionally, 11 ASVs were identified as core ASVs for digesta samples (REF-DID and IM-DID), which were classified as *Geobacillus* (1 ASV), *Lactobacillus* (3 ASVs), *Mycoplasma* (2 ASVs), *Photobacterium* (3 ASVs), *Streptococcus* (1 ASV) and *Weissella* (1 ASV). Two additional core ASVs were identified for the mucosa samples (REF-DIM and IM-DIM), which were classified as *Brevinema andersonii* and *Spirochaetaceae*, respectively. Six additional core ASVs were identified for fish fed the insect meal diet (IM-DID and IM-DIM), which were classified as *Actinomyces*, *Corynebacterium 1*, *Corynebacterium aurimucosum* ATCC 70097, *Lactobacillales*, *RsaHF23* and *Spirochaetaceae*, respectively. No additional core ASVs were identified for fish fed the reference diet (REF-DID and REF-DIM). Lastly, 308 ASVs were found to be more prevalent in IM-DID than in any other sample type.

### Alpha-diversity

**Fig. 3:**
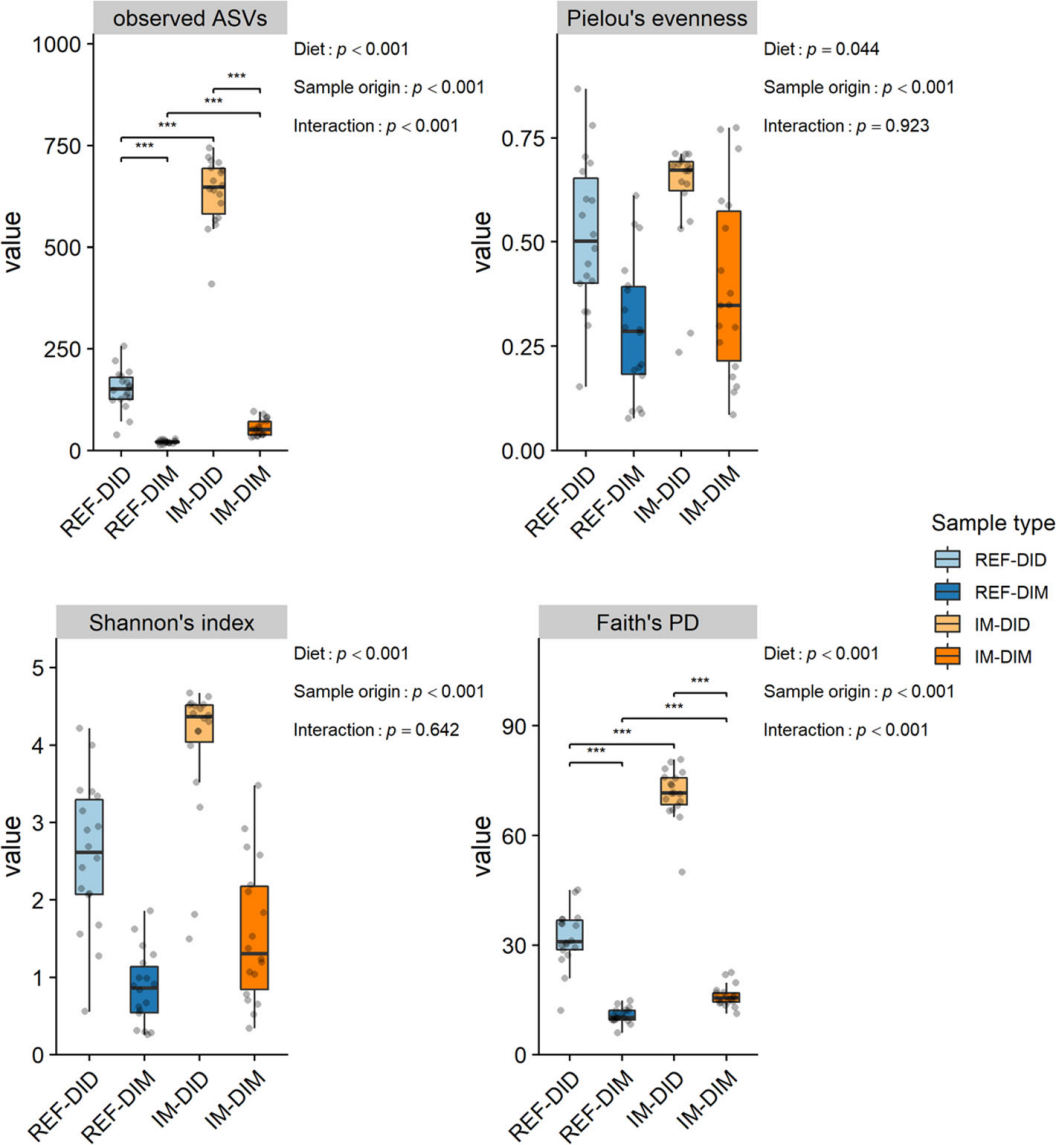
The sample origin and diet effects on the alpha-diversity of distal intestinal microbiota in seawater phase Atlantic salmon. The *p* value of the main effects and their interaction are displayed on the top-right corner of each sub-plot. Asterisks denote statistically significant differences (*, *p* < 0.05; **, *p* < 0.01; ***, *p* < 0.001). PD, phylogenetic diversity; REF, reference diet; IM, insect meal diet; DID, distal intestine digesta; DIM, distal intestine mucosa

Regardless of diet, all the alpha-diversity indices were higher in digesta samples than mucosa samples (*p* < 0.05) (Fig. 3). Independent of sample origin, all the alpha-diversity indices were higher in fish fed the IM diet than those fed the REF diet (*p* < 0.05). A significant interaction between the diet and sample origin effect was detected for the observed ASVs (*p* < 0.001) and Faith’s phylogenetic diversity (*p* < 0.001), both of which showed a stronger diet effect in digesta samples than mucosa samples.

### Beta-diversity

**Fig. 4:**
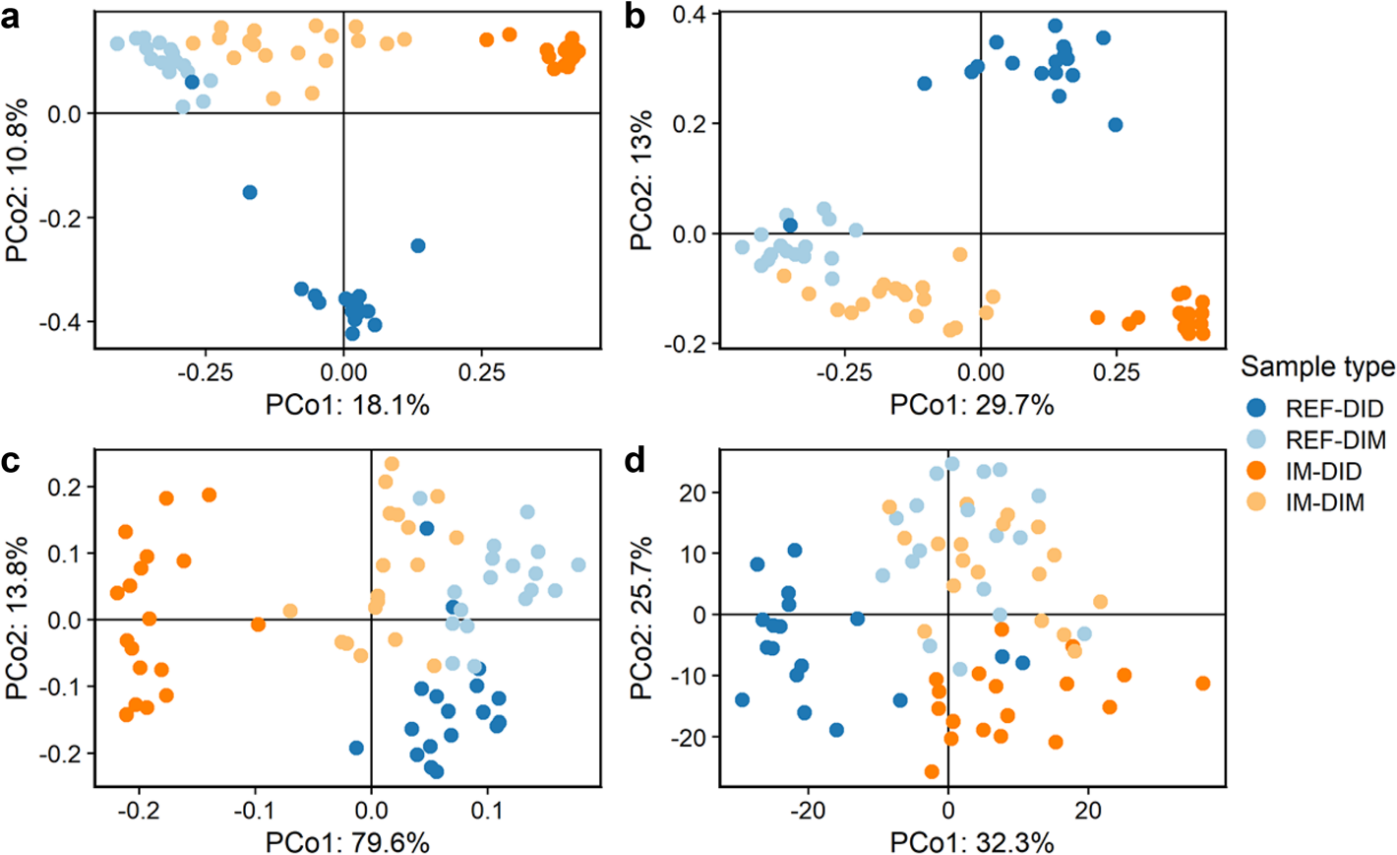
The sample origin and diet effects on the beta-diversity of distal intestinal microbiota in seawater phase Atlantic salmon. The PCoA plots were built on Jaccard (**a**), unweighted UniFrac (**b**), Aitchison (**c**) and phylogenetic isometric log-ratio (PHILR) transformed Euclidean (**d**) distance matrix, respectively. PCo, principle coordinate; REF, reference diet; IM, insect meal diet; DID, distal intestine digesta; DIM, distal intestine mucosa

The PCoA plots built on the Jaccard and unweighted UniFrac distance matrix showed clear separations of samples belonging to different dietary groups and sample origins (Fig. 4a-b). However, the average distance between samples from different dietary groups was dependent on sample origin. Specifically, mucosa samples from different dietary groups formed clusters close to each other, whereas digesta samples from different dietary groups were far apart. The PCoA plots built on the Aitchison and PHILR transformed Euclidean distance matrix also showed separations of samples belonging to different dietary groups and sample origins (Fig. 4c-d). Again, the average distance between samples from different dietary groups was dependent on sample origin. Mucosa samples from different dietary groups formed clusters boarding (Fig. 4c) or overlapping (Fig. 4d) each other, whereas digesta samples from different dietary groups were more clearly separated.

The PERMANOVA and its following conditional contrasts largely confirmed the PCoA results. Regardless of the distance matrix used, both main factors had significant effects on the beta-diversity and their interaction was significant as well (*p* < 0.05) (Table 2). Results on the tests of homogeneity of multivariate dispersions are shown in Table 3. For Jaccard distance, significant differences in the multivariate dispersions were observed between digesta and mucosa samples for both diets (REF-DID VS. REF-DIM, *p* = 0.045; IM-DID VS. IM-DIM, *p* = 0.002), and between diets for digesta samples (REF-DID VS. IM-DID, *p* = 0.002). For unweighted UniFrac distance, IM-DID showed lower multivariate dispersions than other sample types resulting in significant differences compared to REF-DID (*p* = 0.002) and IM-DIM (*p* = 0.002). For Aitchison distance, REF-DIM showed lower multivariate dispersions than other sample types resulting in significant differences compared to REF-DID (*p* = 0.046) and IM-DIM (*p* = 0.046). For PHILR transformed Euclidean distance, the differences in the multivariate dispersions among the sample types were not significant (*p* > 0.05).

### Significant associations between microbial clades and sample metadata

**Fig. 5:**
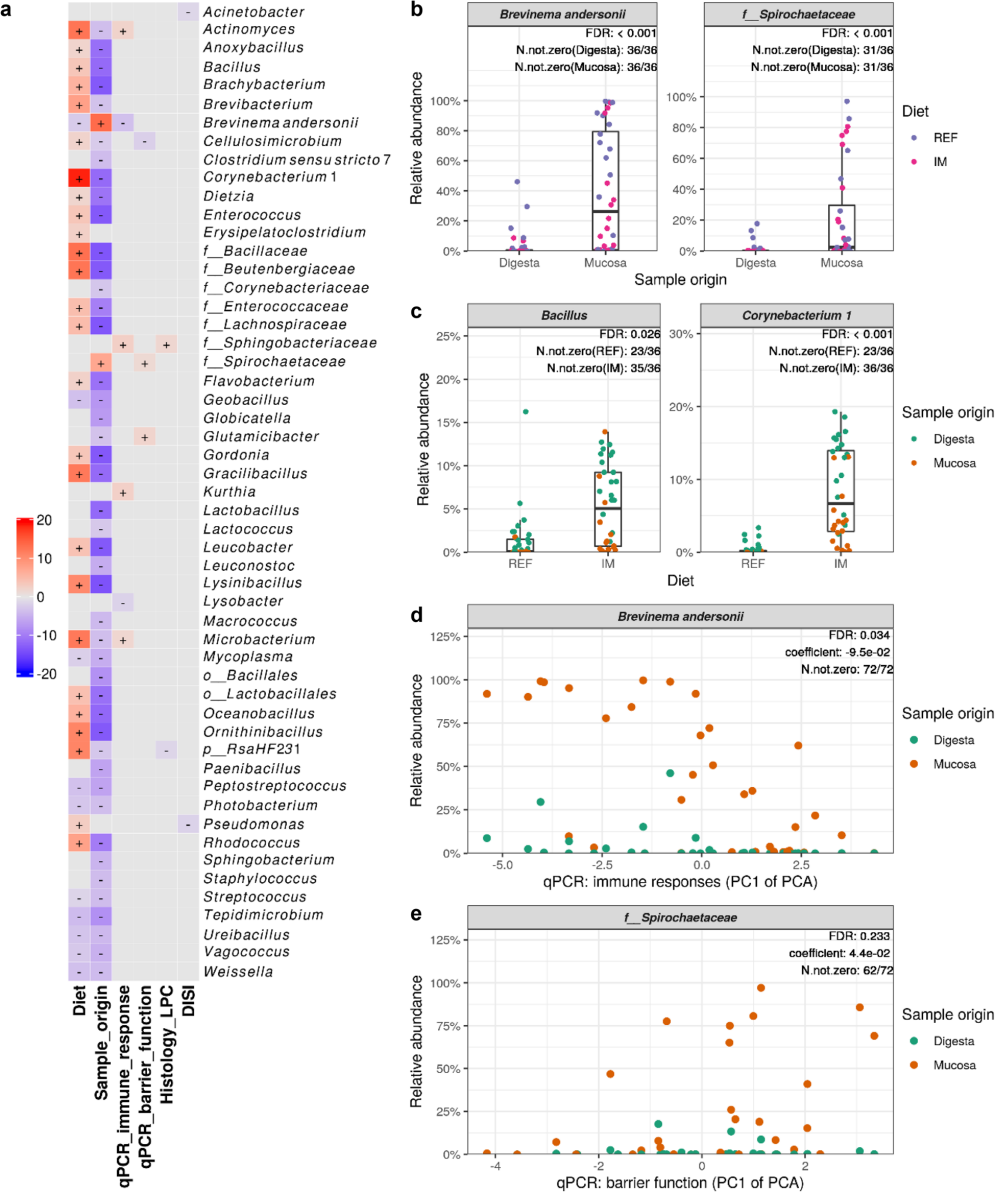
Significant associations between microbial clades and sample metadata. **a** Heatmap summarizing all the significant associations between microbial clades and sample metadata. Color key: -log(*q*-value) * sign (coefficient). Cells that denote significant associations are colored (red or blue) and overlaid with a plus (+) or minus (−) sign that indicates the direction of association: Diet (+), higher abundance in salmon fed the IM diet; Sample_origin (+), higher abundance in mucosa samples; Histology_LPC (+), higher abundance in salmon scored abnormal regarding lamina propria cellularity (LPC) in the distal intestine; DISI (+), positive correlation between microbial clade abundance and distal intestine somatic index (DISI); qPCR_immune_response (+) / qPCR_barrier_function (+), negative correlation between microbial clade abundance and the gene expression levels. **b** Taxa that are more abundant in the intestinal mucosa than the digesta. **c** Representative taxa showing increased relative abundances in both intestinal digesta and mucosa of salmon fed the IM diet. **d** The positive correlation between the relative abundance of *Brevinema andersonii* and immune gene expression levels in the distal intestine. Note that the expression levels of the immune genes decreased as the PC1 of the PCA increased. **e** The negative correlation between the relative abundance of the *Spirochaetaceae* and the expression levels of barrier function relevant genes. Also note that the expression levels of the barrier function relevant genes decreased as the PC1 of the PCA increased. *p*__, phylum; *o*__, order; *f*__, family; FDR, false discovery rate; N.not.zero, number of observations that are not zero; REF, reference diet; IM, insect meal diet

The multivariate association analysis identified 53 taxa showing significant associations with the metadata of interest (Fig. 5a). The diagnostic plots showing the raw data underlying the significant associations are shown in Figs. S3–8. Forty-seven differentially abundant taxa were identified for the sample origin effect, 45 of which, including *Bacillus*, *Enterococcus*, *Flavobacterium*, *Lactobacillus*, *Lactococcus*, *Leuconostoc*, *Mycoplasma*, *Peptostreptococcus*, *Photobacterium*, *Staphylococcus*, *Streptococcus*, *Vagococcus* and *Weissella*, showed lower relative abundances in the mucosa than the digesta (Fig. S3). In contrast, two taxa belonging to the *Spirochaetes* phylum*, Brevinema andersonii* and *Spirochaetaceae*, were enriched in the mucosa (Fig. 5b). Thirty-six differentially abundant taxa were identified for the diet effect, 26 of which showed increased relative abundances in fish fed the IM diet (Fig. S4). Among these 26 taxa, some were enriched in both intestinal digesta and mucosa which included *Actinomyces*, *Bacillaceae*, *Bacillus*, *Beutenbergiaceae*, *Brevibacterium*, *Corynebacterium 1*, *Enterococcus*, *Lactobacillales*, *Microbacterium*, *Oceanobacillus* and *RsaHF231* (partially illustrated as Fig. 5c). For the histological scores, the relative abundance of *Sphingobacteriaceae* and *RsaHF231* were found to increase and decrease, respectively, in fish scored abnormal regarding lamina propria cellularity (LPC) in distal intestine (Fig. S5). The relative abundance of *Acinetobacter* and *Pseudomonas* were negatively correlated with the distal intestine somatic index (DISI) (Fig. S6). Six taxa, including *Actinomyces*, *Brevinema andersonii*, *Kurthia*, *Lysobacter*, *Microbacterium* and the *Sphingobacteriaceae*, were found to associate with the expression of genes related to immune responses (Fig. S7). Notably, the relative abundance of *Brevinema andersonii* showed a clear positive correlation with the expression levels of immune genes (Fig. 5d), which decreased as the PC1 of the PCA increased. Furthermore, 3 taxa including *Cellulosimicrobium*, *Glutamicibacter* and the *Spirochaetaceae* were found to associate with the expression of genes related to barrier functions (Fig. S8). The relative abundance of the *Spirochaetaceae* showed a negative correlation with the expression levels of barrier function relevant genes (Fig. 5e), which decreased as the PC1 of the PCA increased.

## Discussion

### Core microbiota

In accordance with previous studies in Atlantic salmon [33, 46–51], *Aliivibrio*, *Brevinema andersonii* and *Mycoplasma* were identified as core microbiota in the present study. *Aliivibrio* is commonly found in the seawater phase Atlantic salmon intestine [48–50, 52–56] and has been identified as a core taxon of both wild and captive Atlantic salmon [47, 49, 50]. Provided its common presence in seawater, *Aliivibrio* may have originated from the surrounding water and colonized the intestinal mucosa as Atlantic salmon constantly drink seawater to prevent dehydration in a hyperosmotic environment. Currently, *Aliivibrio* comprises of four closely related species including *Aliivibrio fischeri*, *Aliivibrio logei*, *Aliivibrio salmonicida* and *Aliivibrio wodanis*, which were split from the *Vibrio* genus and reclassified as *Aliivibrio* in 2007 [57]. Strains of *A. fischeri* and *A. logei* have been described as bioluminescent symbionts of certain fishes and squids [58], whereas *A. salmonicida* and *A. wodanis* have been identified as pathogens for Atlantic salmon causing cold-water vibriosis [59] and ‘winter ulcer’ [60], respectively. We identified 7 *Aliivibrio* ASVs in this study, four of which, including the core *Aliivibrio* ASV, were closely related and clustered with unknow *Aliivibrio* species in the reference database. Among the known *Aliivibrio* species, *A. logei* is most closely related to the core *Aliivibrio* ASV, which was also the predominant *Aliivibrio* ASV found in the present study. One of the *Aliivibrio* ASVs, which was detected at very low abundances (< 0.00015%), was closely related to *A. wodanis*. These observations coincide with previous findings in Arctic seawater-farmed Atlantic salmon [54], suggesting that *Aliivibrio* in the salmon intestine mostly comprises of commensal species*.*

Though *Spirochaetes* has typically been found in low abundances in the Atlantic salmon intestine [32, 36, 40, 52, 61], two recent studies have identified *Brevinema andersonii* as a core taxon of both digesta- and mucosa-associated intestinal microbiota in seawater phase Atlantic salmon [48, 49]. Notably, *Brevinema andersonii* is also a predominant taxon in the digesta and mucosa in one of the studies [49]. *Brevinema andersonii* was initially isolated from short-tailed shrews (*Blarina brevicauda*) and white-footed mice (*Peromyscus leucopus*) as an infectious pathogen [62]. This taxon has also been found in the intestine and gill tissue of rainbow trout (*Oncorhynchus mykiss*) [63], and intestinal digesta of Senegalese sole (*Solea senegalensis*) [64].

*Mycoplasma* is widely distributed in nature and well known for its minute size and lack of cell wall. It seems to be particularly well-adapted to Atlantic salmon intestine [65]. Like *Aliivibrio*, it has been frequently identified as a core taxon of both wild and captive Atlantic salmon [33, 46, 48–51]. Notably, it was found to be more abundant in marine adults than in freshwater juvenile Atlantic salmon [50] and sporadically predominate intestinal microbial community in the digesta [33, 49, 50, 54, 66] and mucosa [48] reaching > 90% of total reads in extreme cases. Due to its small compact genome and limited biosynthesis capacities, *Mycoplasma* typically forms obligate parasitic or commensal relationships with its host to obtain necessary nutrients such as amino acids, fatty acids and sterols [67]*.* Recent shotgun-metagenomic sequencing of the Atlantic salmon *Mycoplasma* revealed that it is closely related to *Mycoplasma penetrans* [33, 68]. It was suggested that the presence of riboflavin encoding genes and lack of pathogenicity factors in the metagenome-assembled *Mycoplasma* genome is indicative of a symbiotic relationship between the *Mycoplasma* and Atlantic salmon [68].

### Sample origin effect

In line with previous findings in mammals and fish [32, 35–38], we observed substantial differences between digesta- and mucosa-associated microbiota. The microbial richness and diversity were much higher in the digesta than the mucosa, as previously observed in seawater phase Atlantic salmon [32, 36, 49]. Furthermore, most of the bacterial taxa in the distal intestine, including those commonly found in the Atlantic salmon intestine such as *Bacillus*, *Enterococcus*, *Flavobacterium*, *Lactobacillus*, *Lactococcus*, *Leuconostoc*, *Mycoplasma*, *Peptostreptococcus*, *Photobacterium*, *Staphylococcus*, *Streptococcus*, *Vagococcus* and *Weissella*, were less abundant in the mucosa than in the digesta. These results are suggestive of a selection pressure from the host that determines which microbial clades colonize and flourish in the intestinal mucus layer [69]. In this study, two taxa belonging to the *Spirochaetes* phylum, *Brevinema andersonii* and *Spirochaetaceae*, were more abundant in the distal intestine mucosa than the digesta. As aforementioned, *Spirochaetes* were typically found in low abundances in the Atlantic salmon intestine. Yet a recent study also showed that irrespective of diets *Brevinema andersonii* seemed to be more abundant in the intestinal mucosa than the digesta of seawater phase Atlantic salmon [49]. Known for high motility and chemotactic attraction to mucin, some *Spirochaetes* can penetrate the mucus and associate with the intestinal mucosa [70–72]. Further work is required to confirm whether these taxa are consistently enriched in the intestinal mucus layer of seawater phase Atlantic salmon.

### Diet effect

Diet is one of the key factors in shaping the fish intestinal microbiota. In agreement with previous findings in rainbow trout [42, 73, 74] and laying hens [75, 76], we found that the insect meal diet altered the distal intestinal microbiota assemblage resulting in higher microbial richness and diversity. Our findings, showing that the insect meal diet increased the relative abundance of *Actinomyces, Bacillus, Brevibacterium, Corynebacterium 1 and Enterococcus,* are in accord with recent studies in rainbow trout fed diets containing 30% BSF larvae meal [42, 74]. Importantly, these results were partly confirmed in other studies employing fluorescence in situ hybridization for targeted profiling of changes in the intestinal microbiota. Specifically, increased absolute abundance of *Lactobacillus*/*Enterococcus* was found in rainbow trout fed 20% dietary BSF larvae meal [77], whereas increased absolute abundance of *Bacillus*, *Enterococcus* and *Lactobacillus* was documented in Siberian sturgeon (*Acipenser baerii*) fed 15% BSF larvae meal [78].

The increases in the relative abundance of specific microbial clades in Atlantic salmon fed the insect meal diet may be explained by feed-borne microbiota and/or feed composition. Bacterial taxa, including *Actinomyces*, *Bacillus*, *Brevibacterium*, *Corynebacterium*, *Enterococcus*, *Oceanobacillus* and *RsaHF231*, have been found in BSF whole larvae or larvae intestine [79–82]. The fact that *RsaHF231* has not been documented in fish before indicates that these bacterial taxa may have partially originated from BSF larvae meal. Our results from the freshwater feeding trial showed that these bacterial taxa were also enriched in the intestinal digesta and mucosa of Atlantic salmon smolts fed an insect meal diet containing 60% soldier fly larvae meal. Importantly, these bacterial taxa were also detected in the feed pellets which contained considerable amount of bacterial DNA (unpublished data). Given the hydrothermal treatments the feed pellets underwent during the extrusion, the feed-borne microbiota profiled by the DNA sequencing techniques could have largely originated from dead bacteria and bacterial spores rather than living bacteria. As sequencing-based methods cannot differentiate between living and dead cells, future studies should investigate to what extent the feed-borne microbiota may contribute to, or confound the observed diet effects on intestinal microbiota, using methods that distinguish living and dead bacteria such as viability PCR and RNA sequencing [83]. On the other hand, unique nutrients in the insect meal diet such as chitin, an essential component of the insect exoskeleton, may have selectively promoted the growth of certain intestinal microbes. Many bacterial species belonging to *Bacillus* can produce chitinase [84]. *Bacillus* and *Lactobacillus* were two of the predominant taxa in the intestinal mucosa of Atlantic salmon fed a 5% chitin diet, the former of which displayed the highest in vitro chitinase activity [85].

### Significant interactions between diet and sample origin effect

We observed in the present study that the diet effect on the intestinal microbial community richness and structure was dependent on the sample origin, with mucosa-associated intestinal microbiota showing higher resilience to the dietary change. Our results corroborate previous findings in rainbow trout revealing that mucosa-associated intestinal microbiota was less influenced by dietary inclusion of 30% BSF larvae meal compared to digesta-associated intestinal microbiota [41, 42]. Results from molecular-based studies on salmonid intestinal microbiota hitherto suggest that diet modulates digesta- and mucosa-associated intestinal microbiota to varying degrees with the latter generally being more resilient to dietary interventions [32, 39–42, 48]. As such, current practices of profiling only one of or a mixture of digesta- and mucosa-associated microbiota may obscure the response of intestinal microbiota to dietary changes. To fully unveil the response of intestinal microbiota to dietary changes, we recommend concurrent profiling of digesta- and mucosa-associated intestinal microbiota whenever it is feasible.

### Significant associations between microbial clades and sample metadata

To our knowledge, only a few studies have carried out association analysis between intestinal microbial clades and host responses in Atlantic salmon. As such, our results should be treated as preliminary observations and critically evaluated in later studies. Herein, we highlight the significant associations between two mucosa-enriched taxa and host gene expressions in the intestine. Specifically, *Brevinema andersonii*, part of the core microbiota, was associated with the expression of genes related to pro- and anti-inflammatory responses whereas the *Spirochaetaceae* was associated with the expression of genes related to barrier function. Intestinal microbiota is well known to modulate the local immune responses and intestinal epithelial barrier function [86]. Furthermore, it is hypothesized that mucosa-associated microbiota plays a more crucial role in shaping the host immunity in that it can interact both directly and indirectly with intestinal epithelial barrier whereas digesta-associated microbiota can only interact indirectly [69]. Taken together, further research should be undertaken to investigate the potential ecological and functional significance of these two taxa for seawater phase Atlantic salmon.

### Quality control: use of mock and negative controls

As in any field of research, conducting a well-controlled microbiome study requires great care in the experiment design such as setting up appropriate experimental controls. The use of mock as a positive control allows for critical evaluation and optimization of microbiota profiling workflow. That all the bacterial taxa in the mock were correctly identified at the genus level indicates that the current workflow is reliable for the taxonomic profiling of intestinal microbiota. Furthermore, the taxonomic profile of mock from different DNA extraction batches was fairly similar, suggesting that the results generated by the current workflow are also largely reproducible. However, the low concordance between the expected and observed relative abundance of bacterial taxa in the mock is reminiscent of the fact that bias is introduced at different steps of the marker-gene survey [87–89], among which DNA extraction and PCR amplification are the two largest sources of bias due to preferential extraction and amplification of some microbial clades over others. In line with previous observations that Gram-positive bacteria may be more subjective to incomplete lysis during DNA extraction due to their tough cell walls [90, 91], the recovery of most Gram-positive bacteria in the mock was lower than the expected. The insufficient lysing of Gram-positive bacteria in the mock was largely mitigated in our later experiments by using a mixture of beads with different sizes for the bead beating during DNA extraction (unpublished data). The bias in the marker-gene sequencing experiments, as reflected in the observed taxonomic profile of the mock, highlights the necessity of validating such results by absolute quantification techniques such as cultivation (if possible), qPCR, flow cytometry and fluorescence in situ hybridization.

Reagent contamination is a common issue in molecular-based studies of microbial communities. The main contaminating taxon identified in this study is *Pseudomonas*, which has been reported as a common reagent contaminant in numerous studies [92–98]. Given the dominance of *Pseudomonas* in the negative controls of both DNA extraction and PCR, most of the observed contamination has likely derived from PCR reagents such as molecular-grade water [99–101]*.* Notably, *Pseudomonas* has also been isolated from intestinal digesta and mucosa of Atlantic salmon by traditional culturing approaches [85, 102–104], and reported as a member of Atlantic salmon core microbiota in culture-independent studies [32, 36, 46, 47, 51, 105]. Due to the low taxonomic resolution of amplicon sequencing, it is difficult to discern contaminating taxa from true signals solely based on taxonomic labels. The inclusion of negative controls, coupled with quantifications of microbial DNA concentration in the samples, has enabled fast and reliable identification of contaminating taxa in this study. Besides *Pseudomonas*, other common reagent contaminants, including *Bradyrhizobium*, *Burkholderia*, *Comamonas*, *Methylobacterium*, *Propionibacterium*, *Ralstonia*, *Sphingomonas* and *Stenotrophomonas* [97, 99, 101, 106–110], have also been frequently reported as members of Atlantic salmon intestinal microbiota, indicating that existing studies of Atlantic salmon intestinal microbiota may have been plagued with reagent contamination that is hard to ascertain due to lack of negative controls. As reagent contamination is unavoidable, study-specific and can critically influence sequencing-based microbiome analyses [99, 111, 112], negative controls should always be included and sequenced in microbiome studies especially when dealing with low microbial biomass samples like intestinal mucosa.

## Conclusions

In summary, we confirmed previous findings in mammals and fish that intestinal digesta and mucosa harbor microbial communities with clear differences. Regardless of diet, microbial richness and diversity were much higher in the digesta than the mucosa. The insect meal diet altered the distal intestinal microbiota assemblage resulting in higher microbial richness and diversity. The diet effect was however dependent on the sample origin, with mucosa-associated intestinal microbiota being more resilient to the dietary change. To fully unveil the response of intestinal microbiota to dietary changes, concurrent profiling of digesta- and mucosa-associated intestinal microbiota is recommended whenever feasible. Lastly, we identified two mucosa-enriched taxa, *Brevinema andersonii* and *Spirochaetaceae*, which were associated with the expression in the distal intestine of genes related to immune responses and barrier function, respectively. As mucosa-associated microbiota could play a more critical role in shaping the host metabolism, their potential functional significance for seawater phase Atlantic salmon merits further investigations.

## Methods

### Experimental fish, diet and sampling

A 16-week seawater feeding trial with Atlantic salmon (initial body weight = 1.40 kg, S.D. = 0.043 kg) was conducted at the Gildeskål Research Station (GIFAS), Nordland, Norway. The experimental fish were randomly assigned into 6 adjacent square net pens (5 × 5 m) with a depth of 5 m, each containing 90 fish. The fish were fed, in triplicate net pens, either a commercially-relevant reference diet (REF) with a combination of fish meal, soy protein concentrate, pea protein concentrate, corn gluten and wheat gluten as the protein source, or an insect meal diet (IM) wherein all the fish meal and most of the pea protein concentrate were replaced by insect meal produced from BSF larvae. Formulation and proximate composition of the experimental diets are shown in Table 1. The diets were formulated to be isonitrogenous (39% crude protein), isolipidic (29% crude lipid) and isoenergetic (25 MJ/kg DM gross energy), and to meet the nutrient requirements of Atlantic salmon. The diets were extruded, dried and vacuum coated with oils, producing feed pellets with a diameter size of 3.5 mm (Cargill, Dirdal, Norway). The insect meal was produced by Protix Biosystems BV (Dongen, The Netherlands). The fly larvae were grown on feed substrates containing seaweed (*Ascophyllum nodosum*) and vegetable wastes (60:40). After 8 days of growing, the larvae were harvested and partially defatted before being dried and ground to make the insect meal. The insect meal contains about 52% crude protein and 18% crude lipid. Fish were fed by hand until apparent satiation once or twice daily depending on the duration of daylight. During the feeding trial, the water temperature was 8.3 ± 3.7 °C, dissolved oxygen 8.9 ± 1 mg/L and salinity 31.6 ± 0.8 ‰. Further details on the nutritional composition of the insect meal and diets have been reported elsewhere [45].

**Table 1:** Formulation and proximate composition of the experimental diets

	REF	IM
**Ingredients (% wet-weight)**		
Fishmeal LT94	10	0
Black soldier fly larva meal^a^	0	14,75
Soy protein concentrate	25	25
Corn gluten meal	7,5	7,5
Wheat gluten meal	3,35	6,88
Pea protein concentrate 55	8,8	2,84
Fish oil	10,18	14,76
Rapeseed oil	20,95	14,73
Binder	12,32	11,24
Additives	1,89	2,29
**Chemical composition (wet-weight basis)**		
Dry matter (%)	93	95
Crude Protein (%)	38	39
Crude Lipid (%)	29	29
Ash (%)	4,6	4,5
Carbohydrates (%)	11,6	11,4
Gross energy (MJ/kg)	24,6	25
TBARS (nmol/g)	3	4,9

*REF* reference diet, *IM* insect meal diet, *TBARS* Thiobarbituric acid reactive substances

^a^Partially defatted. Crude protein: 52%, crude lipid: 18%. Produced by the Protix Biosystems BV (Dongen, The Netherlands)

**Table 2:** PERMANOVA results and subsequent conditional contrasts

Distance matrix	Main effects		Interaction	Conditional contrasts			
	Diet	Sample origin		REF-DID VS. IM-DID	REF-DIM VS. IM-DIM	REF-DID VS. REF-DIM	IM-DID VS. IM-DIM
Jaccard	0.001	0.001	0.001	0.001	0.001	0.001	0.001
Unweighted UniFrac	0.001^a^	0.001	0.001	0.001^a^	0.001	0.001	0.001
Aitchison	0.001	0.003	0.004	0.002	0.004	0.004^a^	0.002^a^
PHILR (Euclidean)^b^	0.001	0.001	0.001	0.001	0.005	0.001	0.001

*REF* reference diet, *IM* insect meal diet, DID distal intestine digesta, *DIM* distal intestine mucosa

^a^Monte Carlo *p* value

^b^Phylogenetic isometric log-ratio transformed Euclidean distance

**Table 3:** Test of homogeneity of multivariate dispersions among groups

Distance matrix	Conditional contrasts			
	REF-DID VS. IM-DID	REF-DIM VS. IM-DIM	REF-DID VS. REF-DIM	IM-DID VS. IM-DIM
Jaccard	0.002	0.087	0.045	0.002
Unweighted UniFrac	0.002	0.711	0.200	0.002
Aitchison	0.453	0.046	0.046	0.369
PHILR (Euclidean)^a^	0.240	0.266	0.240	0.266

*REF* reference diet, *IM* insect meal diet, *DID* distal intestine digesta, *DIM* distal intestine mucosa

^a^Phylogenetic isometric log-ratio transformed Euclidean distance

At the termination of the feeding trial, the average body weight of fish reached 3.7 kg. Six fish were randomly taken from each net pen, anesthetized with tricaine methanesulfonate (MS222®; Argent Chemical Laboratories, Redmond, WA, USA) and euthanized by a sharp blow to the head. After cleaning the exterior of each fish with 70% ethanol, the distal intestine, i.e., the segment from the increase in intestinal diameter and the appearance of transverse luminal folds to the anus, was aseptically removed from the abdominal cavity, placed in a sterile Petri dish and opened longitudinally. Only fish with digesta along the whole intestine were sampled to ensure that the intestine had been exposed to the diets. The intestinal digesta was gently scraped and collected into a 50 mL skirted sterile centrifuge tube and mixed thoroughly using a spatula. An aliquot of the homogenate was then transferred into a 1.5 mL sterile Eppendorf tube and snap-frozen in liquid N_2_ for the profiling of digesta-associated intestinal microbiota. A tissue section from the mid part of the distal intestine was excised and rinsed in sterile phosphate-buffered saline 3 times to remove traces of the remaining digesta. After rinsing, the intestinal tissue was longitudinally cut into 3 pieces for histological evaluation (fixed in 4% phosphate-buffered formaldehyde solution for 24 h and transferred to 70% ethanol for storage), RNA extraction (preserved in RNAlater solution and stored at − 20 °C) and profiling of mucosa-associated intestinal microbiota (snap-frozen in liquid N_2_), respectively. The collection of microbiota samples was performed near a gas burner to secure aseptic conditions. After the sampling of each fish, tools were cleaned and decontaminated by a 70% ethanol spray and flaming. Microbiota samples of the distal intestine digesta (DID) and mucosa (DIM) were transported in dry ice and stored at − 80 °C until DNA extraction.

### DNA extraction

Total DNA was extracted from ~ 200 mg distal intestine digesta or mucosa using the QIAamp® DNA Stool Mini Kit (Qiagen, Hilden, Germany; catalog no., 51,504) with some modifications to the manufacturer’s specifications as described before [32], except that 2 mL prefilled bead tubes (Qiagen; catalog no., 13,118–50) were used for the bead beating. For quality control purposes, a companion “blank extraction” sample was added to each batch of sample DNA extraction by omitting the input material, whereas an additional microbial community standard (ZymoBIOMICS™, Zymo Research, California, USA; catalog no., D6300), i.e. mock, was included for each DNA extraction kit as a positive control. The mock consists of 8 bacteria (*Pseudomonas aeruginosa*, *Escherichia coli*, *Salmonella enterica*, *Lactobacillus fermentum*, *Enterococcus faecalis*, *Staphylococcus aureus*, *Listeria monocytogenes*, *Bacillus subtilis*) and 2 yeasts (*Saccharomyces cerevisiae*, *Cryptococcus neoformans*).

### Amplicon PCR

The V1–2 hypervariable regions of the bacterial 16S rRNA gene were amplified using the primer set 27F (5′-AGA GTT TGA TCM TGG CTC AG-3′) and 338R (5′-GCW GCC WCC CGT AGG WGT-3′) [113]. The PCR was run in a total reaction volume of 25 μL containing 12.5 μL of Phusion® High-Fidelity PCR Master Mix (Thermo Scientific, CA, USA; catalog no., F531L), 10.9 μL molecular grade H_2_O, 1 μL DNA template and 0.3 μL of each primer (10 μM). The amplification program was set as follows: initial denaturation at 98 °C for 3 min; 35 cycles of denaturation at 98 °C for 15 s, annealing decreasing from 63 °C to 53 °C in 10 cycles for 30 s followed by 25 cycles at 53 °C for 30 s, and extension at 72 °C for 30 s; followed by a final extension at 72 °C for 10 min. For samples with faint or invisible bands in the agarose gel after PCR, the PCR condition was optimized by applying serial dilutions to the DNA templates to reduce the influence of PCR inhibitors. All the digesta samples were diluted 1:2 in buffer ATE (10 mM Tris-Cl, pH 8.3, with 0.1 mM EDTA and 0.04% NaN3) whereas all the mucosa samples were diluted 1:32. The formal amplicon PCR was run in duplicate incorporating two negative PCR controls, which were generated by replacing the template DNA with molecular grade H_2_O. The duplicate PCR products were then pooled and examined by a 1.5% agarose gel electrophoresis.

### Quantification of 16S rRNA gene by qPCR

To assist in identifying contaminating sequences, the 16S rRNA gene quantity in the diluted DNA templates used for the amplicon PCR was measured by qPCR. The qPCR assays were performed using a universal primer set (forward, 5′-CCA TGA AGT CGG AAT CGC TAG-3′; reverse, 5′-GCT TGA CGG GCG GTG T-3′) that has been used for bacterial DNA quantification in previous studies [114, 115]. The assays were carried out using the LightCycler 96 (Roche Applied Science, Basel, Switzerland) in a 10 μL reaction volume, which contained 2 μL of PCR-grade water, 1 μL diluted DNA template, 5 μL LightCycler 480 SYBR Green I Master Mix (Roche Applied Science) and 1 μL (3 μM) of each primer. Samples, together with the extraction blanks and mock, were run in duplicate in addition to Femto™ bacterial DNA standards (Zymo Research; catalog no., E2006) and a no-template control of the qPCR assay. The qPCR program encompassed an initial enzyme activation step at 95 °C for 2 min, 45 three-step cycles of 95 °C for 10 s, 60 °C for 30 s and 72 °C for 15 s, and a melting curve analysis at the end. Quantification cycle (Cq) values were determined using the second derivative method [116]. The specificity of qPCR amplification was confirmed by evaluating the melting curve of qPCR products and the band pattern on the agarose gel after electrophoresis. The inter-plate calibration factor was calculated following the method described in [117], using the bacterial DNA standards as inter-plate calibrators.

### Sequencing

The sequencing was carried out on a Miseq platform following the Illumina 16S metagenomic sequencing library preparation protocol [118]. Briefly, the PCR products were cleaned using the Agencourt AMPure XP system (Beckman Coulter, Indiana, USA; catalog no., A63881), multiplexed by dual indexing using the Nextera XT Index Kit (Illumina, California, USA; catalog no., FC-131-1096) and purified again using the AMPure beads. After the second clean-up, representative libraries were selected and analyzed using the Agilent DNA 1000 Kit (Agilent Technologies, California, USA; catalog no., 5067–1505) to verify the library size. Cleaned libraries were quantified using the Invitrogen Qubit™ dsDNA HS Assay Kit (Thermo Fisher Scientific, California, USA; catalog no., Q32854), diluted to 4 nM in 10 mM Tris (pH 8.5) and finally pooled in an equal volume. Negative controls with library concentrations lower than 4 nM were pooled in equal volume directly. Due to the low diversity of amplicon library, 15% Illumina generated PhiX control (catalog no., FC-110-3001) was spiked in by combining 510 μL amplicon library with 90 μL PhiX control library. The library was loaded at 6 pM and sequenced using the Miseq Reagent Kit v3 (600-cycle) (Illumina; catalog no., MS-102-3003).

### Sequence data processing

The raw sequence data were processed by the DADA2 1.14 in R 3.6.3 [119] to infer amplicon sequence variants (ASVs) [120]. Specifically, the demultiplexed paired-ended reads were trimmed off the primer sequences (forward reads, first 20 bps; reverse reads, first 18 bps), truncated at the position where the median Phred quality score crashed (forward reads, at position 290 bp; reverse reads, at position 248 bp) and filtered off low-quality reads. After trimming and filtering, the run-specific error rates were estimated and the ASVs were inferred by pooling reads from all the samples sequenced in the same run. The chimeras were removed using the “pooled” method after merging the reads. The resulting raw ASV table and representative sequences were imported into QIIME2 (version, 2020.2) [121]. The taxonomy was assigned by a scikit-learn naive Bayes machine-learning classifier [122], which was trained on the SILVA 132 99% OTUs [123] that were trimmed to only include the regions of 16S rRNA gene amplified by our primers. ASVs identified as chloroplasts or mitochondria were excluded from the ASV table. The ASV table was conservatively filtered to remove ASVs that had no phylum-level taxonomic assignment or appeared in only one biological sample. Contaminating ASVs were identified based on two suggested criteria: contaminants are often found in negative controls and inversely correlate with sample DNA concentration [98]. The ASVs filtered from the raw ASV table were also removed from the representative sequences, which were then inserted into a reference phylogenetic tree built on the SILVA 128 database using SEPP [124]. The alpha rarefaction curves and the core metrics results were generated with a sampling depth of 10,000 and 2047 sequences per sample, respectively (Fig. S9). For downstream data analysis and visualization, QIIME2 artifacts were imported into R using the *qiime2R* package [125] and a *phyloseq* [126] object was assembled from the sample metadata, ASV table, taxonomy and phylogenetic tree. The core ASVs were calculated using a prevalence threshold at 80% and visualized by the Venn’s diagram. The alpha-diversity indices, including observed ASVs, Pielou’s evenness, Shannon’s index and Faith’s phylogenetic diversity (PD), were computed via the R packages *microbiome* [127] and *picante* [128]. For beta-diversity analyses, we used distance matrices including Jaccard distance, unweighted UniFrac distance, Aitchison distance and phylogenetic isometric log-ratio (PHILR) transformed Euclidean distance. Since rarefying remains to be the best solution for unweighted distance matrices [129], the Jaccard distance and unweighted UniFrac distance were computed in QIIME2 using the rarefied ASV table. The compositionality-aware distance matrices, Aitchison distance and PHILR transformed Euclidean distance, were calculated using the unrarefied ASV table. The Aitchison distance was computed by the *DEICODE* plugin in QIIME2, a form of Aitchison distance that is robust to high levels of sparsity by using the matrix completion to handle the excessive zeros in the microbiome data [130]. The PHILR transform of the ASV table was performed in R using the *philr* package [131]. The selected distance matrices were explored and visualized by the principal coordinates analysis (PCoA).

### Multivariate association analysis

To reduce the multiple testing burden, the ASV table was collapsed at the genus level before running the multivariate association analysis. Bacterial taxa of very low abundance (< 0.01%) or low prevalence (present in < 25% of samples) were removed from the feature table. The microbial clades were then tested for significant associations with metadata of interest by MaAsLin2 (version, 0.99.12) (https://huttenhower.sph.harvard.edu/maaslin2) in R, using the default parameters. The results of the analysis are the associations of specific microbial clades with metadata, deconfounding the influence of other factors included in the model. Association was considered significant when the *q*-value was below 0.25. Metadata included in the multivariate association testing are fixed factors Diet + Sample origin + distal intestine somatic index (DISI) + lamina propria cellularity (histological scores) + immune response (qPCR) + barrier function (qPCR), and random factors FishID + NetPen. FishID was nested in NetPen, and NetPen nested in Diet. The methodological approach to these parameters was reported in a previous study [44]. Lamina propria cellularity reflects the severity of inflammation in the distal intestine. Based on the degree of cellular infiltration within the lamina propria, a value of normal, mild, moderate, marked or severe was assigned. To make the data appropriate for the association testing, the highly skewed five-category scores were collapsed into more balanced binary data, i.e., normal and abnormal. The immune-related genes included for the association testing were myeloid differentiation factor 88 (*myd88*), interleukin 1β (*il1β*), interleukin 8 (*il8*), cluster of differentiation 3 γδ (*cd3γδ*), transforming growth factor β1 (*tgfβ1*), interferon γ (*ifnγ*), interleukin 17A (*il17a*), fork-head box P3 (*foxp3*) and interleukin 10 (*il10*), whose expression levels were higher in the distal intestine of fish assigned abnormal regarding lamina propria cellularity. Since the expression levels of immune-related genes were highly correlated, we ran a principal component analysis (PCA) and extracted the first principle component (PC1) for the association testing to avoid multicollinearity and reduce the number of association testing. For genes relevant to the barrier function, which included claudin-15 (*cldn15*), claudin-25b (*cldn25b*), zonula occludens 1 (*zo1*), E-cadherin / cadherin 1 (*cdh1*) and mucin-2 (*muc2*), we also used the PC1 of the PCA for the association testing based on the same considerations.

### Statistics

All the statistical analyses were run in R except for the PERMANOVA, which was run in PRIMER (version 7; PRIMER-e). The differences in the alpha-diversity indices were compared using linear mixed-effects models via the *lme4* package [132]. Predictor variables in the models included the fixed effects Diet + Sample origin + Diet x Sample origin, and the random effects FishID + NetPen. The models were validated by visual inspections of residual diagnostic plots generated by the *ggResidpanel* package [133]. The statistical significance of fixed predictors was estimated by Type III *ANOVA* with Kenward-Roger’s approximation of denominator degrees of freedom via the *lmerTest* package [134]. When the interaction between the main effects was significant, conditional contrasts for the main effects were made via the *emmeans* package [135]. To compare the differences in beta-diversity, we performed the PERMANOVA [136] using the same predictors included in the linear mixed-effects models. Terms with negative estimates for components of variation were sequentially removed from the model via term pooling, starting with the one showing the smallest mean squares. At each step, the model was reassessed whether more terms needed to be removed or not. Conditional contrasts for the main effects were constructed when their interaction was significant. Monte Carlo *p* values were computed as well when the unique permutations for the terms in the PERMANOVA were small (< 100). The homogeneity of multivariate dispersions among groups was visually assessed with boxplots and was formally tested by the permutation test, PERMDISP [137], via the R package *vegan* [138]. Multiple comparisons were adjusted by the Benjamini-Hochberg procedure where applicable. Differences were regarded as significant when *p* < 0.05.

## Supplementary Information

**Additional file 1: Figure S1.** Quantification of bacterial 16S rRNA gene in different sample types using qPCR. Since the Cq values of most mucosa-associated samples were out of the linear range of the standard curve, the Cq value was used as a proxy of 16S rRNA gene quantity which is reliable for the screening of contaminant sequences. Data are presented as mean ± 1 standard deviation overlaying the raw data points. Abbreviations: REF, reference diet; IM, insect meal diet; DID, distal intestine digesta; DIM, distal intestine mucosa. **Figure S2.** Taxonomic profile of the mock (A) and contaminating features in the negative controls (B). The lowest level of taxonomic ranks was displayed for each taxon. EB, extraction blank; LB, library blank. **Figure S3**. Microbial clades showing significant associations with sample origin. *p*__, phylum; *o*__, order; *f*__, family; FDR, false discovery rate; N.not.zero, number of non-zero observations; REF, reference diet; IM, insect meal diet. **Figure S4.** Microbial clades showing significant associations with diet. *p*__, phylum; *o*__, order; *f*__, family; FDR, false discovery rate; N.not.zero, number of non-zero observations; REF, reference diet; IM, insect meal diet. **Figure S5.** Microbial clades showing significant associations with histological scores on lamina propria cellularity in the distal intestine. *p*__, phylum; *f*__, family; FDR, false discovery rate; N.not.zero, number of non-zero observations. **Figure S6.** Microbial clades showing significant associations with distal intestine somatic index (DISI). FDR, false discovery rate; N.not.zero, number of non-zero observations. **Figure S7.** Microbial clades showing significant associations with immune gene expressions in the distal intestine. Since the expression levels of immune genes were highly correlated, we ran a principle component analysis (PCA) and used the first principle component (PC1) for the association testing to avoid multicollinearity and reduce the number of association testing. Note that the expression levels of immune genes decrease as the PC1 increases from left to right. Hence, a positive correlation coefficient denotes a negative association between the microbial clade and immune gene expressions, and vice versa. *f*__, family; FDR, false discovery rate; N.not.zero, number of non-zero observations. **Figure S8.** Microbial clades showing significant associations with expressions of barrier function related genes in the distal intestine. Since the expression levels of barrier function related genes were highly correlated, we ran a principle component analysis (PCA) and used the first principle component (PC1) for the association testing to avoid multicollinearity and reduce the number of association testing. Note that the expression levels of barrier function related genes decrease as the PC1 increases from left to right. Hence, a positive correlation coefficient denotes a negative association between the microbial clade and barrier function related gene expressions, and vice versa. *f*__, family; FDR, false discovery rate; N.not.zero, number of non-zero observations. **Figure S9.** Rarefaction curves based on Observed ASVs for the different sample types. The rarefaction analysis showed that mucosa samples (REF-DIM, IM-DIM) reached the saturation phase at a subsampling depth of 2000 sequences whereas digesta samples (REF-DID, IM-DID) reached the saturation phase at a subsampling depth of 9500 sequences. To preserve a maximum number of samples for the downstream data analysis, we rarefied the ASV table to 2047 sequences per sample which left out 2 samples. To ensure that the subsampling depth of 2047 sequences per sample produced reliable comparisons of microbial communities, we computed compositionality-aware distance matrices, the Aitchison distance and PHILR transformed Euclidean distance, which do not require rarefying and use all the sequences in the samples.

**Additional file 2: Table S1.** Contaminating features removed from the ASV table.

**Additional file 3: Table S2.** The prevalence of core ASVs in different sample types.

## Data Availability

The raw 16S rRNA gene sequencing data are deposited at the NCBI SRA database under the BioProject PRJNA555355. Other raw data and code for reproducing our results are available from the GitHub repository (https://github.com/yanxianl/Li_AqFl2-Microbiota_ASM_2020).

## Abbreviations

ASVs: amplicon sequence variants
BSF: black soldier fly
Cq: quantification cycle
DID: distal intestine digesta
DIM: distal intestine mucosa
DISI: distal intestine somatic index
IM: insect meal
LPC: lamina propria cellularity
PC1: principal component one
PCA: principal component analysis
PCoA: principal coordinates analysis
PD: phylogenetic diversity
PERMANOVA: permutational multivariate analysis of variance
PHILR: phylogenetic isometric log-ratio
REF: reference
