# Supplementary material for: Differential response of digesta- and mucosa-associated intestinal microbiota to dietary insect meal during the seawater phase of Atlantic salmon
Source: Anim Microbiome. 2021 Jan 7;3:8. doi: 10.1186/s42523-020-00071-3 (PMC7934271; doi:10.1186/s42523-020-00071-3)
Supplement: Supplementary file 1 — Additional file 1: Figure S1. Quantification of bacterial 16S rRNA gene in different sample types using qPCR. Since the Cq values of most mucosa-associated samples were out of the linear range of the standard curve, the Cq value was used as a proxy of 16S rRNA gene quantity which is reliable for the screening of contaminant sequences. Data are presented as mean ± 1 standard deviation overlaying the raw data points. Abbreviations: REF, reference diet; IM, insect meal diet; DID, distal intestine digesta; DIM, distal intestine mucosa. Figure S2. Taxonomic profile of the mock (A) and contaminating features in the negative controls (B). The lowest level of taxonomic ranks was displayed for each taxon. EB, extraction blank; LB, library blank. Figure S3. Microbial clades showing significant associations with sample origin. p__, phylum; o__, order; f__, family; FDR, false discovery rate; N.not.zero, number of non-zero observations; REF, reference diet; IM, insect meal diet. Figure S4. Microbial clades showing significant associations with diet. p__, phylum; o__, order; f__, family; FDR, false discovery rate; N.not.zero, number of non-zero observations; REF, reference diet; IM, insect meal diet. Figure S5. Microbial clades showing significant associations with histological scores on lamina propria cellularity in the distal intestine. p__, phylum; f__, family; FDR, false discovery rate; N.not.zero, number of non-zero observations. Figure S6. Microbial clades showing significant associations with distal intestine somatic index (DISI). FDR, false discovery rate; N.not.zero, number of non-zero observations. Figure S7. Microbial clades showing significant associations with immune gene expressions in the distal intestine. Since the expression levels of immune genes were highly correlated, we ran a principle component analysis (PCA) and used the first principle component (PC1) for the association testing to avoid multicollinearity and reduce the number of association testing. Note [file 42523_2020_71_MOESM1_ESM.docx]

# **
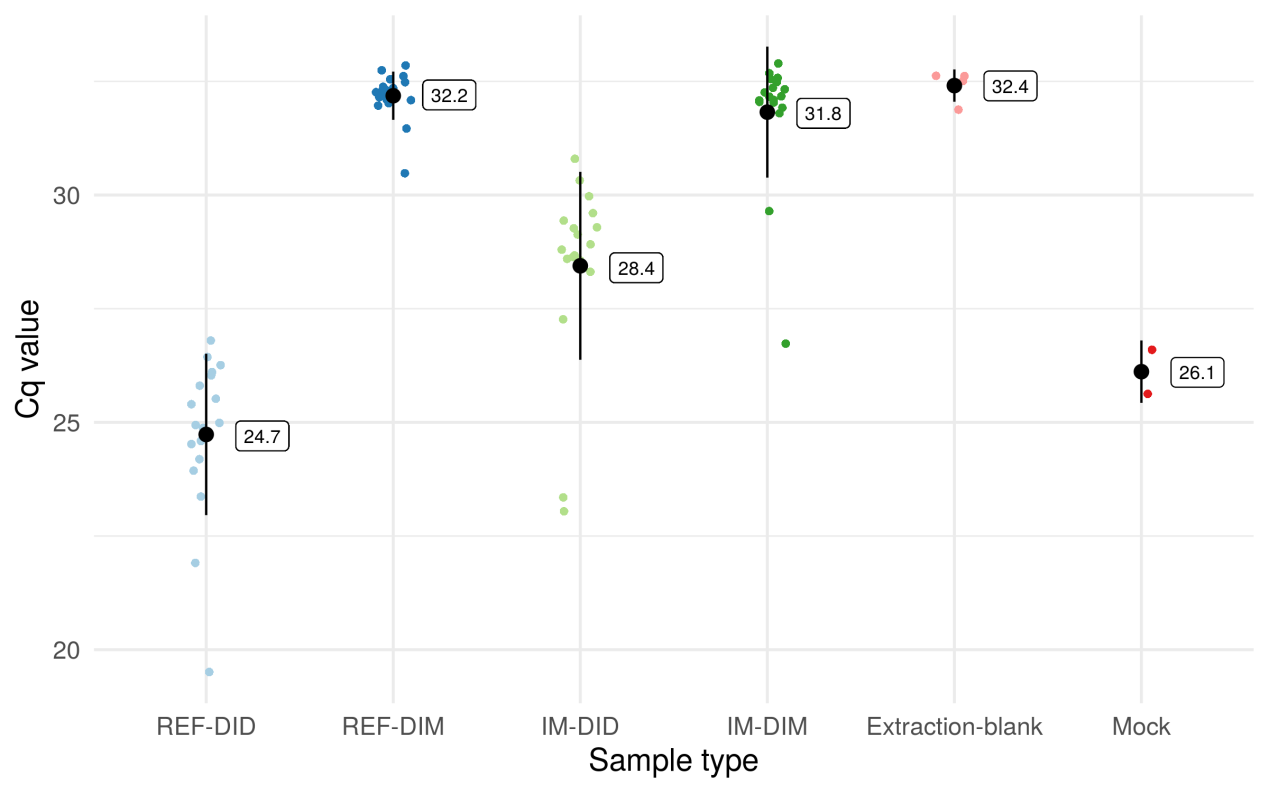
**Supplemental figures

**Figure S1. Quantification of bacterial 16S rRNA gene in different sample types using qPCR.** Since the Cq values of most mucosa-associated samples were out of the linear range of the standard curve, the Cq value was used as a proxy of 16S rRNA gene quantity which is reliable for the screening of contaminant sequences. Data are presented as mean ± 1 standard deviation overlaying the raw data points. Abbreviations: REF, reference diet; IM, insect meal diet; DID, distal intestine digesta; DIM, distal intestine mucosa.


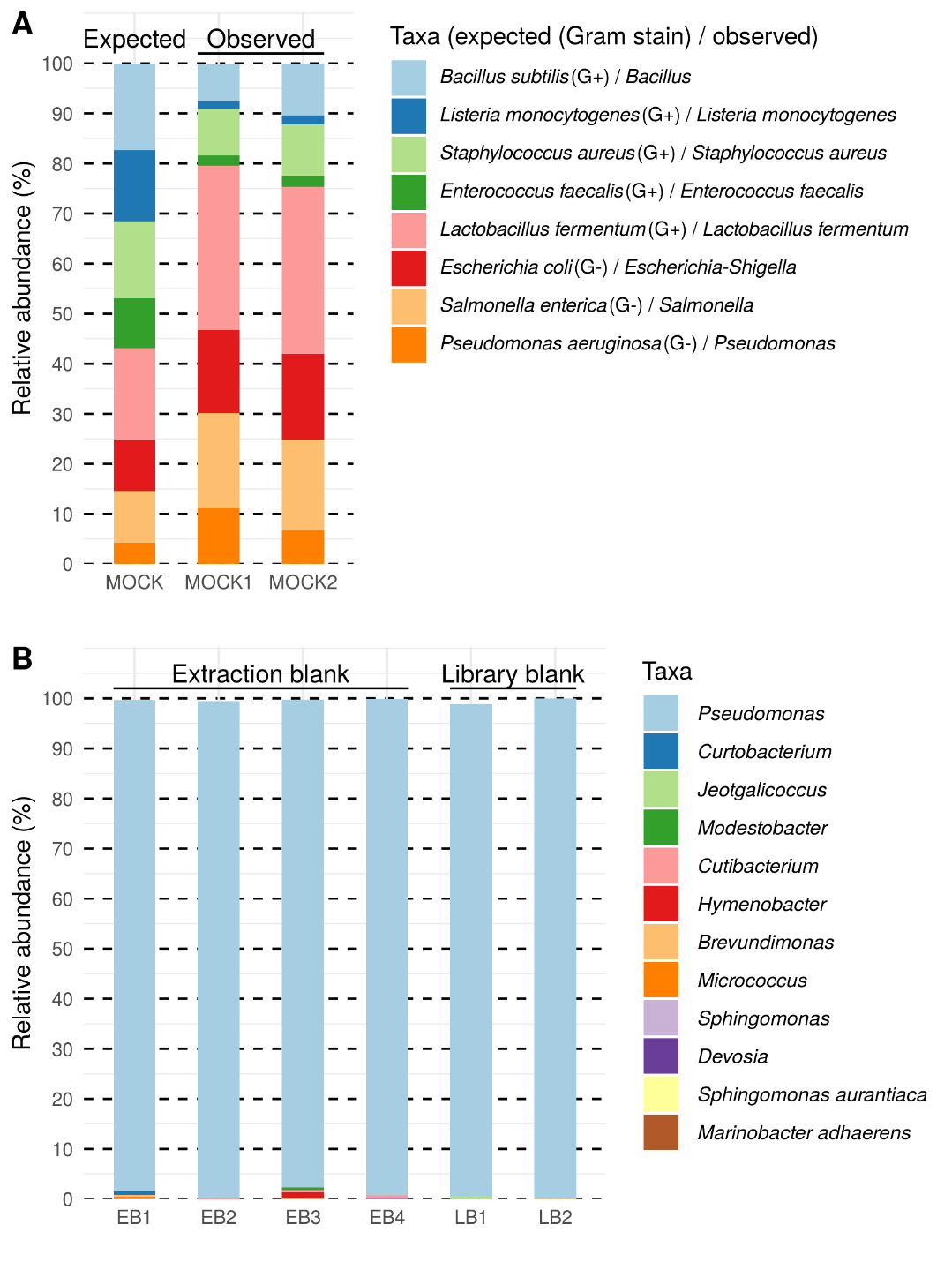
**Figure S2. Taxonomic profile of the mock (A) and contaminating features in the negative controls (B).** The lowest level of taxonomic ranks was displayed for each taxon. EB, extraction blank; LB, library blank.

**
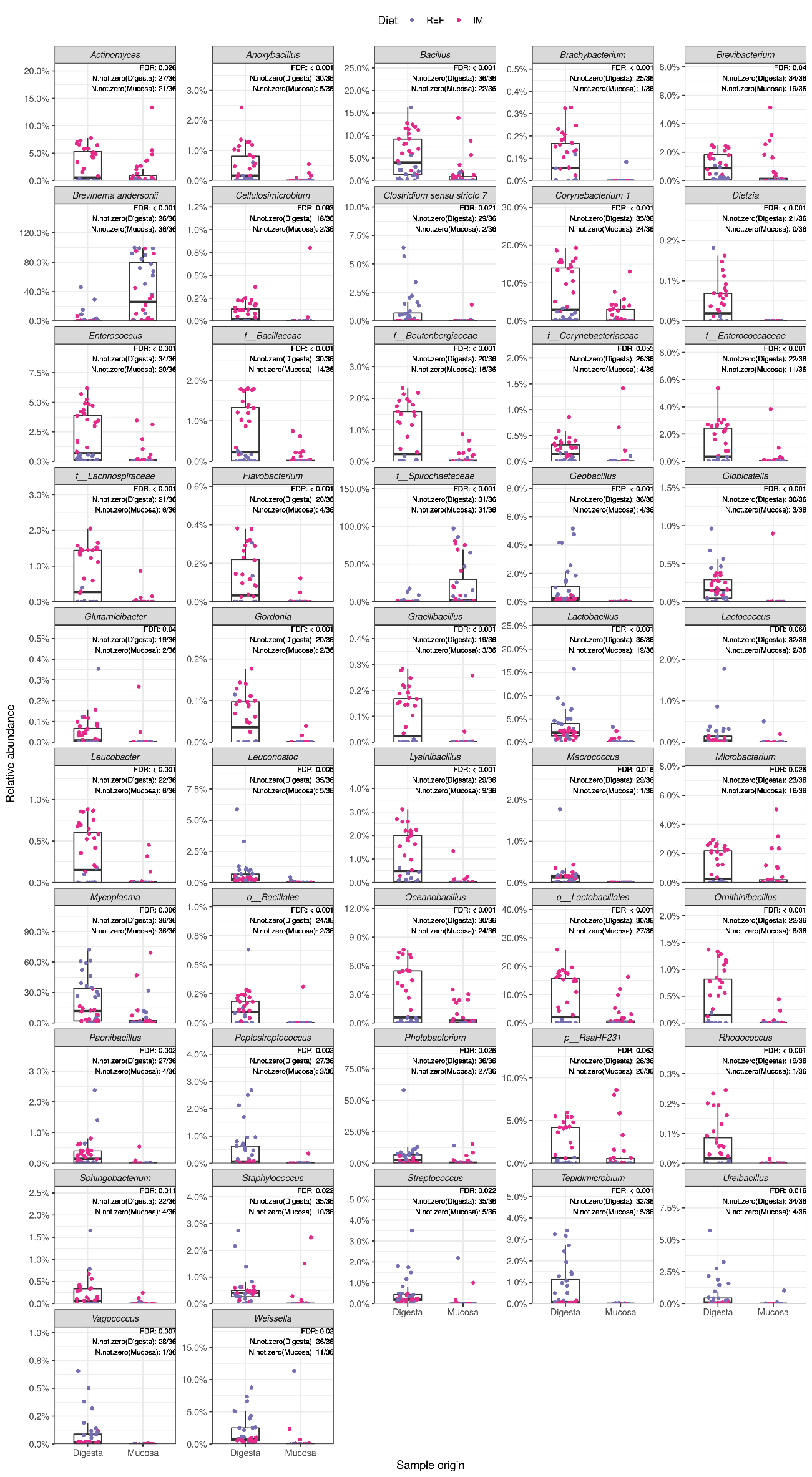
Figure S3. Microbial clades showing significant associations with sample origin.** *p*__, phylum; *o*__, order; *f*__, family; FDR, false discovery rate; N.not.zero, number of non-zero observations; REF, reference diet; IM, insect meal diet.

**
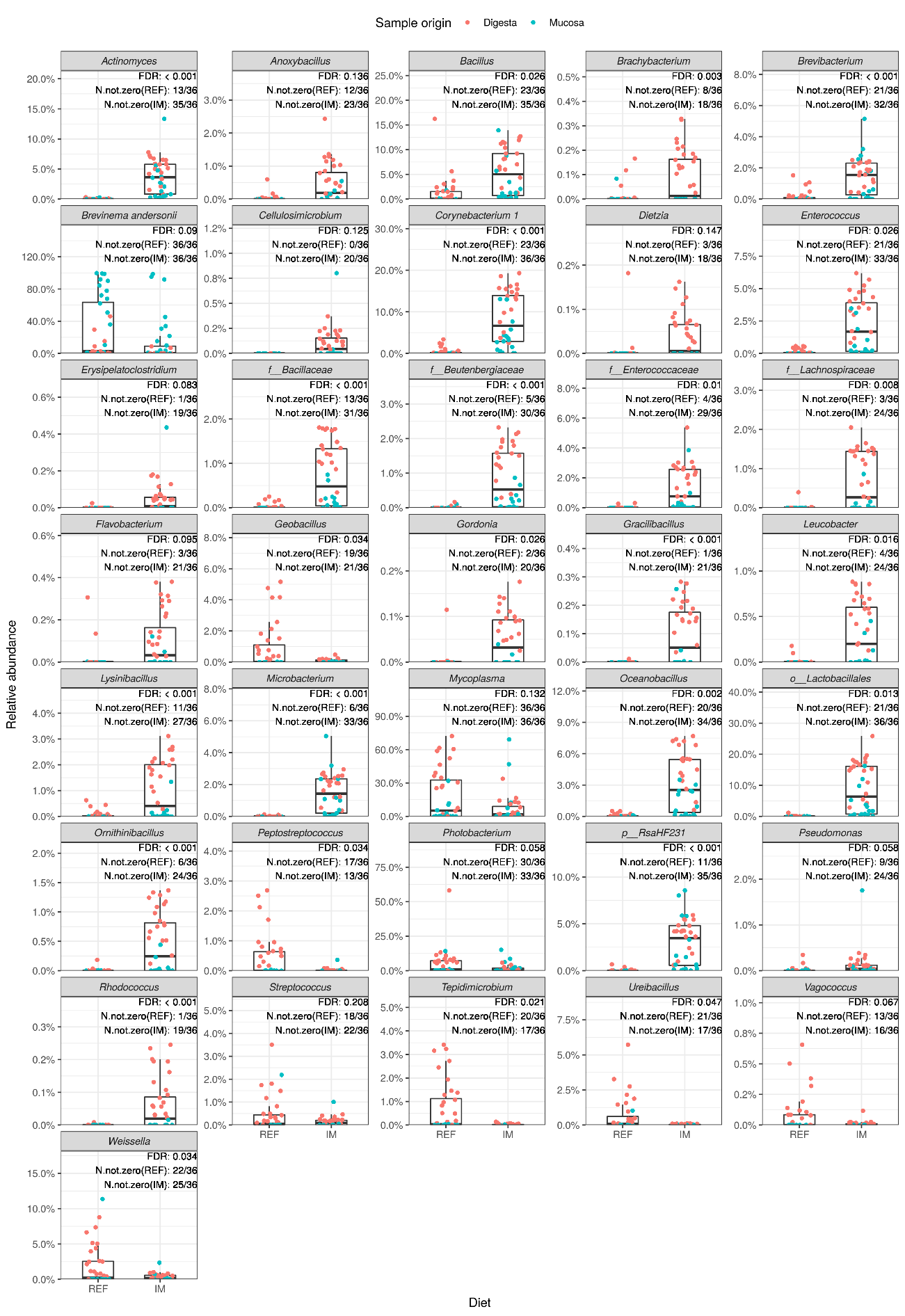
Figure S4. Microbial clades showing significant associations with diet.** *p*__, phylum; *o*__, order; *f*__, family; FDR, false discovery rate; N.not.zero, number of non-zero observations; REF, reference diet; IM, insect meal diet.

**
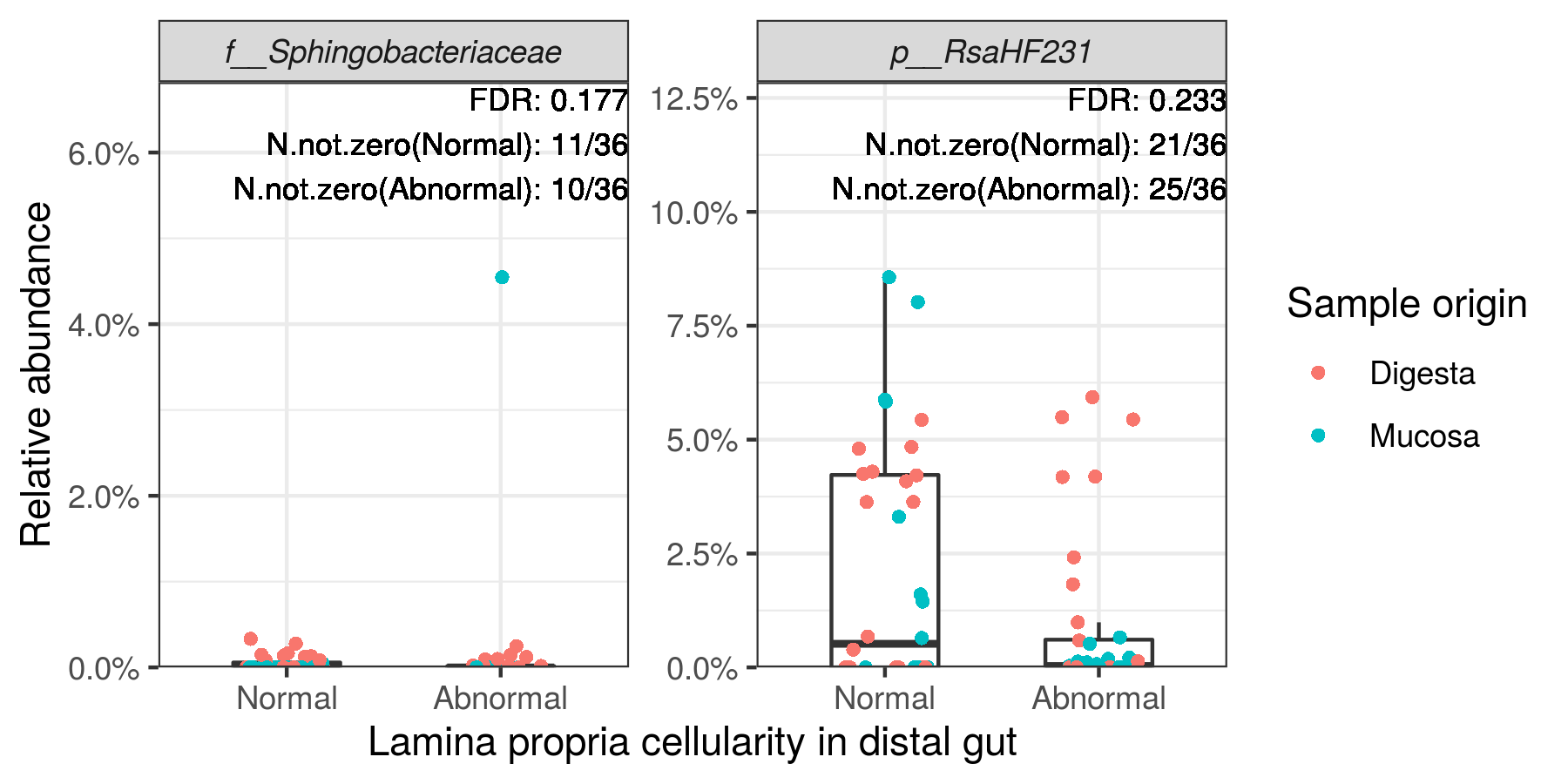
Figure S5. Microbial clades showing significant associations with histological scores on lamina propria cellularity in the distal intestine.** *p*__, phylum; *f*__, family; FDR, false discovery rate; N.not.zero, number of non-zero observations.

**
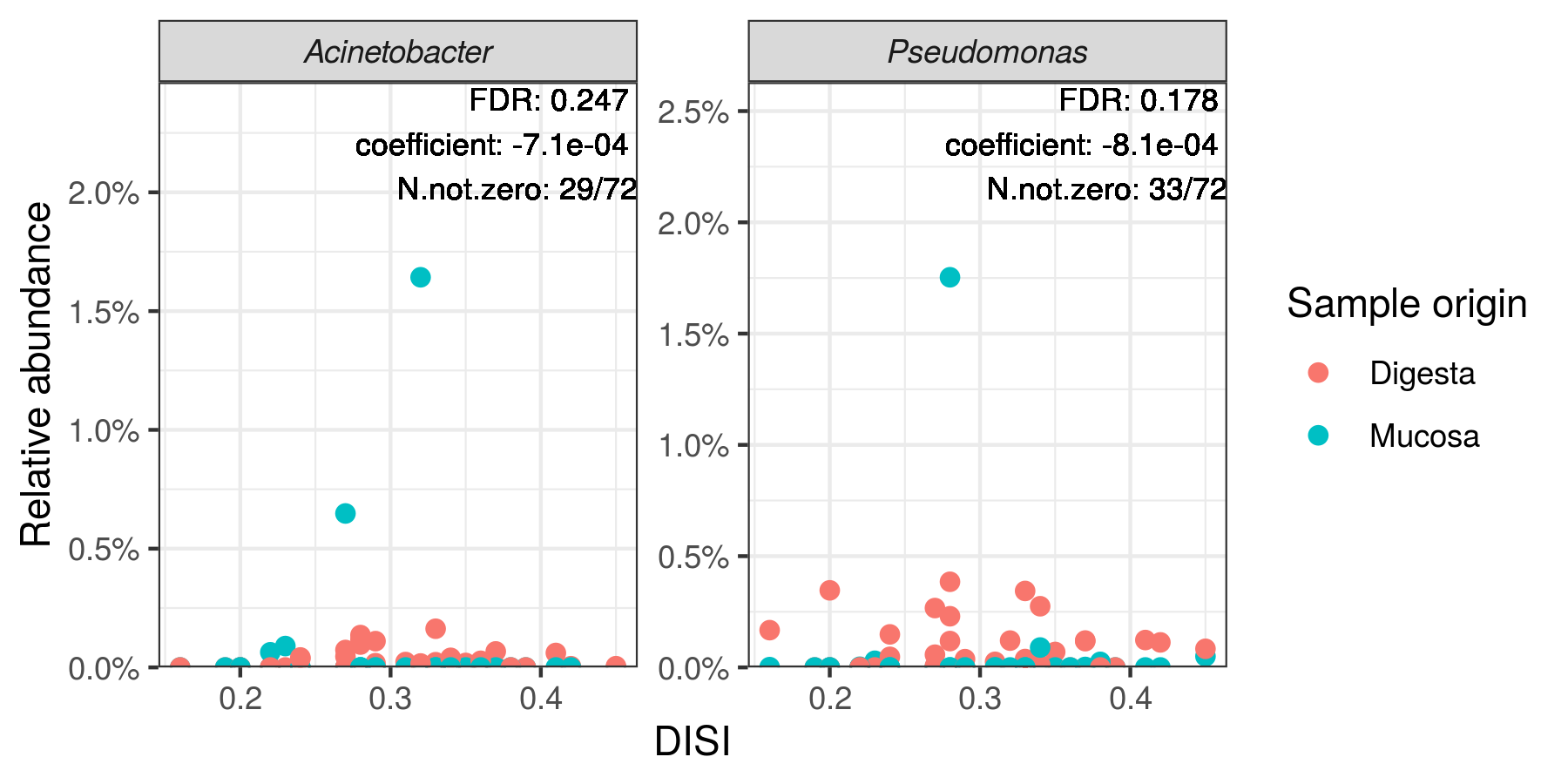
Figure S6. Microbial clades showing significant associations with distal intestine somatic index (DISI).** FDR, false discovery rate; N.not.zero, number of non-zero observations.

**
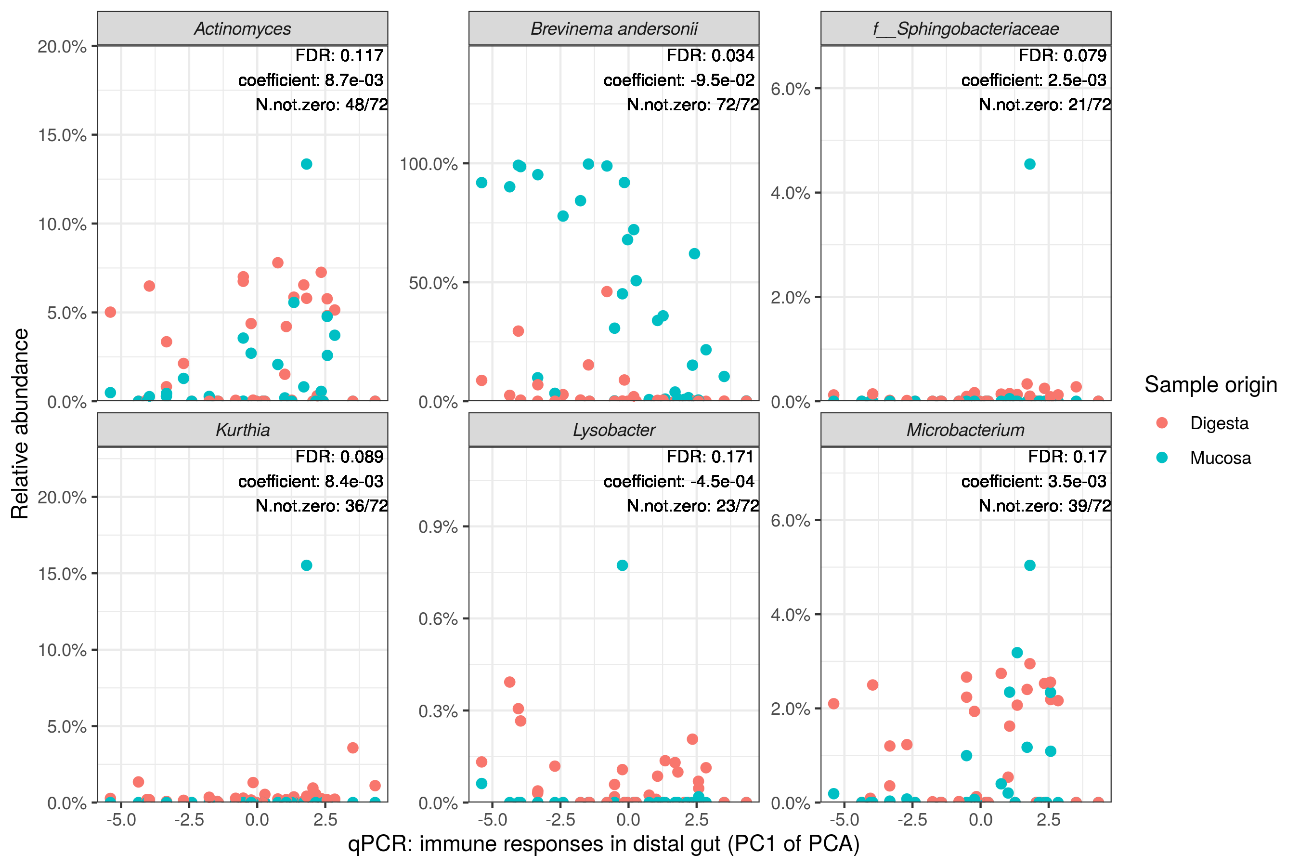
Figure S7. Microbial clades showing significant associations with immune gene expressions in the distal intestine.** Since the expression levels of immune genes were highly correlated, we ran a principle component analysis (PCA) and used the first principle component (PC1) for the association testing to avoid multicollinearity and reduce the number of association testing. Note that the expression levels of immune genes decrease as the PC1 increases from left to right. Hence, a positive correlation coefficient denotes a negative association between the microbial clade and immune gene expressions, and vice versa. *f*__, family; FDR, false discovery rate; N.not.zero, number of non-zero observations.

**
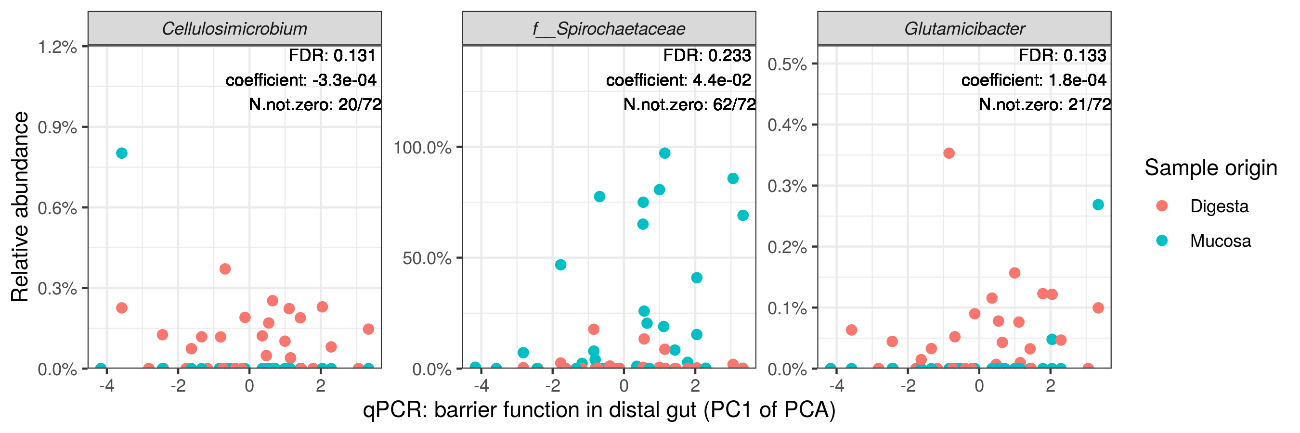
Figure S8. Microbial clades showing significant associations with expressions of barrier function related genes in the distal intestine.** Since the expression levels of barrier function related genes were highly correlated, we ran a principle component analysis (PCA) and used the first principle component (PC1) for the association testing to avoid multicollinearity and reduce the number of association testing. Note that the expression levels of barrier function related genes decrease as the PC1 increases from left to right. Hence, a positive correlation coefficient denotes a negative association between the microbial clade and barrier function related gene expressions, and vice versa. *f*__, family; FDR, false discovery rate; N.not.zero, number of non-zero observations

**
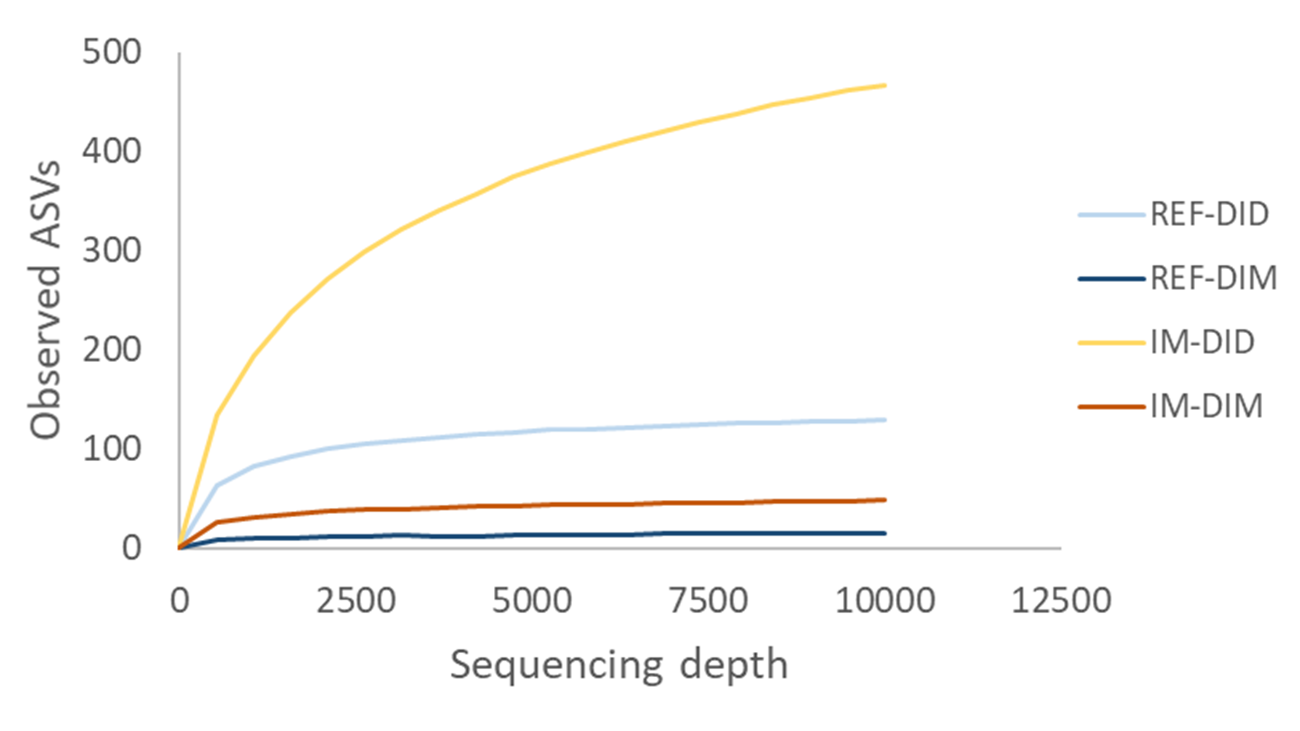
Figure S9. Rarefaction curves based on Observed ASVs for the different sample types.** The rarefaction analysis showed that mucosa samples (REF-DIM, IM-DIM) reached the saturation phase at a subsampling depth of 2000 sequences whereas digesta samples (REF-DID, IM-DID) reached the saturation phase at a subsampling depth of 9500 sequences. To preserve a maximum number of samples for the downstream data analysis, we rarefied the ASV table to 2047 sequences per sample which left out 2 samples. To ensure that the subsampling depth of 2047 sequences per sample produced reliable comparisons of microbial communities, we computed compositionality-aware distance matrices, the Aitchison distance and PHILR transformed Euclidean distance, which do not require rarefying and use all the sequences in the samples.
